# Boron neutron capture therapy in the context of tumor heterogeneity: progress, challenges, and future perspectives

**DOI:** 10.3389/fonc.2025.1601013

**Published:** 2025-10-17

**Authors:** Rongmiao Zhou, Zhun You, Liang Liu

**Affiliations:** Cancer Institute, The Fourth Hospital of Hebei Medical University, Shijiazhuang, Hebei, China

**Keywords:** boron neutron capture therapy (BNCT), tumor heterogeneity, progress, challenges, future directions

## Abstract

Boron neutron capture therapy (BNCT), as an emerging binary treatment method, has shown the advantage of effectively treating tumors while sparing normal tissues, utilizing the characteristics of boron’s nuclear capture and fission reactions, as well as the distinct distribution of boron delivery agents in tumor tissues and healthy tissues. Currently, numerous boron delivery agents have been developed to improve their targeting property, biocompatibility, solubility, and nuclear localization capability. The shift of neutron source from being based on nuclear reactors to being based on accelerators facilitates the conduct of clinical trials. BNCT has demonstrated promising results in treating head and neck cancers, gliomas, and skin melanomas. In addition, researches on the application of BNCT have been carried out in other tumors, such as liver cancer, lung cancer, and breast cancer. Notably, in 2020, BNCT was approved for clinical use in the treatment of unresectable locally advanced or locally recurrent head and neck cancer. Subsequently, post-marketing surveillance confirmed its safety and efficacy. Despite the progress made, BNCT still encounters substantial challenges in enhancing its efficacy. This review provides a comprehensive synthesis of the literature on BNCT over the last decade. It systematically examines the treatment’s mechanism of action, the landscape of clinical research, key markers and factors influencing therapeutic efficacy, and the primary challenges and future directions for the field. The development of BNCT is impeded by several significant challenges, including the research and development of boron delivery agents, the construction of neutron sources, the management of tumor heterogeneity, the advancement of clinical translation, and the securement of economic and logistical support. These challenges can only be systematically overcome through the organic integration of technological innovation, policy support, clinical standardization, and cross-disciplinary collaboration, thereby creating a synergistic effect.

## Introduction

In 2022, there were nearly 20 million new cancer cases worldwide. There are significant regional differences in the occurrence of cancer, with risk factors such as smoking, alcohol consumption, obesity, and infections being associated with cancer development. Cancer is the second most common cause of death globally. In 2022, 9.7 million people died from cancer (1, 2). Cancer poses a significant threat to human health and imposes a substantial economic burden on families and society. Conventional cancer treatments, such as surgery, chemotherapy, and radiotherapy, each have their own limitations. While surgery can remove a large portion of tumor cells, it fails to completely eliminate cancer cells. Chemotherapy often results in severe side effects, compromising patients’ quality of life. Furthermore, prolonged use of chemotherapy can increase the risk of drug resistance and cancer recurrence. Radiotherapy may lead to damage in the irradiated area or nearby normal tissues, causing serious complications. Boron neutron capture therapy (BNCT) is an emerging binary treatment method based on the nuclear capture and fission reactions of boron. In 1935, Taylor et al. discovered that boron-10 nuclei capture neutrons to form unstable boron-11, which subsequently undergoes prompt nuclear fission. This process results in the production of high linear energy transfer (LET) alpha particles (^4^He) and the recoil of lithium-7 (^7^Li) nuclei (3). The movement of these particles roughly spans the diameter of a cell. In 1936, a hypothesis was put forward suggesting the potential use of the abovementioned reaction in treating tumors (4). If boron-10 can selectively accumulate in tumors to reach an effective concentration while remaining at minimal or nonexistent levels in normal tissues, neutron irradiation can then target and eliminate boron-laden tumor cells without harming the surrounding normal tissues. This theory has subsequently attracted extensive attention across disciplines such as nuclear physics, pharmacology, biology, and medicine, prompting a surge in BNCT-related research encompassing cellular, animal, and clinical studies, in multiple countries, including Japan, China, the United States, Germany, Finland, and Sweden, among others (5). Currently, studies have demonstrated that BNCT has promising clinical effectiveness in treating head and neck cancers, brain tumors, and skin melanomas (6–16). In particular, in 2020, BNCT was approved for clinical use in the treatment of locally advanced or recurrent unresectable head and neck cancers in Japan, with treatment expenses covered by medical insurance. By utilizing the distinct distribution of boron in tumor tissues compared with normal tissues and its capacity to selectively eliminate tumor cells via fission reactions upon neutron capture, BNCT has the potential to effectively treat tumors while sparing healthy tissues. However, as a binary therapy, the implementation of BNCT includes multiple steps: the development and administration of boron delivery agents, neutron sources, the determination of boron concentration, the calculation of neutron dose, the selection of patients who can benefit from BNCT, and the evaluation of BNCT efficacy. Therefore, there are still many challenges in improving the efficacy of BNCT. This review synthesizes various studies on BNCT, outlining its mechanism, the state of clinical trials, indicators for assessing its effectiveness, and the obstacles it confronts. This review also discusses how tumor heterogeneity influences the effects of BNCT. The article would provide insights for future BNCT-related research.

## Action mechanism of the BNCT

BNCT inhibits DNA synthesis, induces DNA damage, and subsequently activates certain signaling pathways, which ultimately leads to cell cycle arrest, apoptosis, necrosis, autophagy, or mitotic catastrophe (17, 18). In a hamster cheek pouch oral cancer model, BNCT might help control tumor growth by inhibiting DNA synthesis (19). When faced with BNCT-induced DNA double-strand breaks, thyroid follicular carcinoma cells relied mainly on homologous recombination repair (HRR) to remedy damage, whereas melanoma cells used both nonhomologous end joining (NHEJ) and HRR to address the harm caused by BNCT (20). Upon BNCT treatment, melanoma cells presented decreased cyclin D1 expression, a notable increase in cleaved caspase-3 levels, and a reduction in the mitochondrial membrane potential. Additionally, a decrease in collagen synthesis might make melanoma cells more prone to detach from the extracellular matrix (ECM), potentially inducing cell death, known as anoikis. Hence, cell cycle arrest, apoptosis, and changes in the ECM were involved in the process of BNCT treatment for melanoma (21). In human squamous cell carcinoma SAS cells, BNCT treatment led to the activation of caspase-9 and caspase-3, as well as the cleavage of poly (ADP-ribose) polymerase-1 (PARP-1) and phosphorylated histone H2AX (γH2AX). Proteomic analysis revealed that proteins involved in endoplasmic reticulum functions, DNA repair, and RNA processing played a role in BNCT treatment. Additionally, BNCT induced fragmentation of the endoplasmic reticulum-located lymphocyte-restricted membrane protein (LRMP) in SAS cells and rat tumor graft models, suggesting that the dynamic changes in the LRMP might be involved in the cellular response to BNCT (22). An analysis of the proteins in extracellular vesicles released by SAS cells after BNCT via liquid chromatography coupled with tandem mass spectrometry (LC-MS/MS) suggested that processes such as apoptosis, DNA repair, and inflammatory reactions could be linked to the outcomes of BNCT (23).

To improve the efficacy and safety of BNCT, cellular and animal experiments were conducted to identify biomarkers of response to BNCT. The early response to BNCT is usually evaluated by measuring DNA double-strand breaks and other indicators of damage, including micronuclei formation, the appearance of γH2AX foci, p53 binding protein 1 (53BP1) foci formation, and the presence of poly(ADP-ribose) (20, 24–27). Fragmentation of the endoplasmic reticulum-located LRMP is also a potential marker for BNCT treatment (22). High mobility group box 1 (HMGB1), a protein widely expressed within cells, plays a role in the response to DNA damage and in cell death processes (27). When undergoing apoptosis, autophagy, and necrosis, cells release HMBG1 (28–30). In the early stages of BNCT treatment, the expression of HMBG1 was elevated in lymphosarcoma-bearing rats (27). Following BNCT therapy, elevated levels of HMBG1 were detected in the supernatants of cultured human squamous cell carcinoma SAS and melanoma A375 cells, as well as in the plasma of mice bearing SAS cell xenograft tumors (31). Consequently, HMBG1 might be a potential biomarker for evaluating the response to BNCT.

## Current status of BNCT clinical research

### Head and neck cancers

Head and neck cancers tend to recur following treatment. Clinical trials have been carried out to evaluate the use of BNCT in treating these cancers. For example, a Japanese study between 2001 and 2007 involving 62 patients with head and neck cancers demonstrated that BNCT resulted in a median survival time (MST) of 10.1 months, with a 1-year overall survival (OS) rate of 43.2% (6). Another study conducted between 1999 and 2012 indicated that 76% of the 29 patients who received treatment responded positively to BNCT, with a median progression-free survival (PFS) of 7.5 months (7). These study outcomes highlight the effectiveness of BNCT in managing head and neck cancers. Notably, in 2020, BNCT gained approval in Japan for the clinical treatment of head and neck cancer patients. Postmarketing surveillance demonstrated that BNCT was safe and effective for the treatment of unresectable locally advanced or locally recurrent head and neck cancers (32, 33).

### Brain cancers

Gliomas are common brain tumors. Currently, numerous experiments have been performed to investigate the therapeutic effects of BNCT on gliomas, and the results are very encouraging. Between 2001 and 2005, a total of 42 patients with gliomas in Sweden underwent BNCT treatment. The MSTs for patients with nonrecurrent and recurrent gliomas were 17.7 months and 22.2 months, respectively (8). From 1998 to 2008, a series of studies in Japan investigated the effects of BNCT on glioma patients. Despite variations in drug protocols and radiation dosages across these studies, patients generally exhibited positive outcomes. The reported MSTs ranged from 10.8 to 25.7 months (9–12).

Like gliomas, meningiomas present a challenging disease process to control. BNCT has shown promise in treating meningiomas. A study between 2005 and 2011 involving 20 patients with recurrent high-grade meningioma revealed that BNCT led to improvements in clinical symptoms such as hemiparesis and facial pain, with an MST of 14.1 months (13). Another study with 44 meningioma patients showed that, following BNCT, the patients had an MST of 29.6 months (14).

### Melanoma

Melanoma, a type of cancer that originates from melanocytes, is the most fatal form of skin cancer. The first BNCT experiment for melanoma conducted in Japan indicated a 73% complete response rate among 22 patients (15). A separate study with long-term monitoring of 8 melanoma patients after BNCT revealed an overall control rate of 88% (16). In Argentina, between October 2003 and June 2007, seven patients with multiple cutaneous melanoma located in extremities received eight sessions of BNCT. Among the evaluable nodules, the overall response rate was 69.3% (34). Nonetheless, these studies were limited by their small sample sizes.

### Other cancers

Whether BNCT can be applied to other types of tumors and its effectiveness are also of great interest to researchers. To date, numerous clinical, cellular, and animal studies have explored the therapeutic efficacy of BNCT for tumors beyond the brain, head and neck, and melanoma, including hepatocellular carcinoma, lung cancer, sarcomas, and metastatic tumors. While many excellent reviews have summarized BNCT’s application in the former, more established cancer types, this paper focuses on its use in other tumor types (5, 35–38). As shown in **Supplementary Table 1**, we presented a comprehensive overview of this research, detailing the tumor types investigated, the number of patients or models used, the boron delivery agents and their administration routes, as well as the treatment outcomes and side effects.

## Clinical biomarkers of BNCT

Certain studies have identified markers associated with BNCT, which can aid in identifying suitable candidates for BNCT, monitoring the effectiveness of BNCT, and predicting the prognosis of patients undergoing BNCT. A moderate correlation was identified between L-type amino acid transporter 1 (LAT1) expression and 4-borono-2-¹^8^F-fluoro-phenylalanine (¹^8^F-FBPA) accumulation in a cohort of 28 patients with head and neck cancer. Notably, in cases where a discrepancy existed between LAT1 expression intensity and ^18^F-FBPA accumulation, LAT1 expression proved to be a superior predictor of treatment response to BNCT. Therefore, the LAT1 expression score represented a promising biomarker for predicting the efficacy of BNCT (39). Lin et al. assessed the predictive value of fluorine-18 labeled boronophenylalanine positron emission tomography (^18^F-BPA-PET) for prognosis in patients with malignant brain tumors receiving BNCT. The results showed that for patients who achieved an objective response, the OS of patients with a BPA tumor to normal tissue (T/N) ratio greater than 2.5 was significantly greater than that of patients with a T/N ratio less than 2.5. In other words, patients whose BPA T/N ratio was higher than 2.5 before undergoing BNCT were more likely to achieve an objective response, and those who did so experienced a relatively prolonged OS. In brief, the BPA T/N ratio could be used as a significant criterion for determining suitability for BNCT, with the attainment of an objective response being a key factor affecting the prognosis of patients (40). Five patients with recurrent head and neck cancer post-radiotherapy received a combination therapy of BNCT and fractionated image-guided intensity-modulated radiation therapy (IG-IMRT). A close correlation was observed between circulating monocytic myeloid-derived suppressor cell (M-MDSC) levels and tumor size, assessed at two time points: prior to BNCT treatment and one month after the final IG-IMRT session. The level of circulating M-MDSCs might serve as a biomarker for tumor progression in patients with recurrent head and neck cancer (41). Among 15 patients with advanced malignant brain tumors treated with BNCT, those with circulating M-MDSC levels below 5% exhibited a significantly longer median survival time compared to patients with levels above 5%. The level of circulating M-MDSCs might act as a potential biomarker for predicting the therapeutic efficacy of BNCT in this patient population (42).

## Factors associated with the therapeutic efficacy of BNCT

### Boron delivery agents

**Figure 1:**
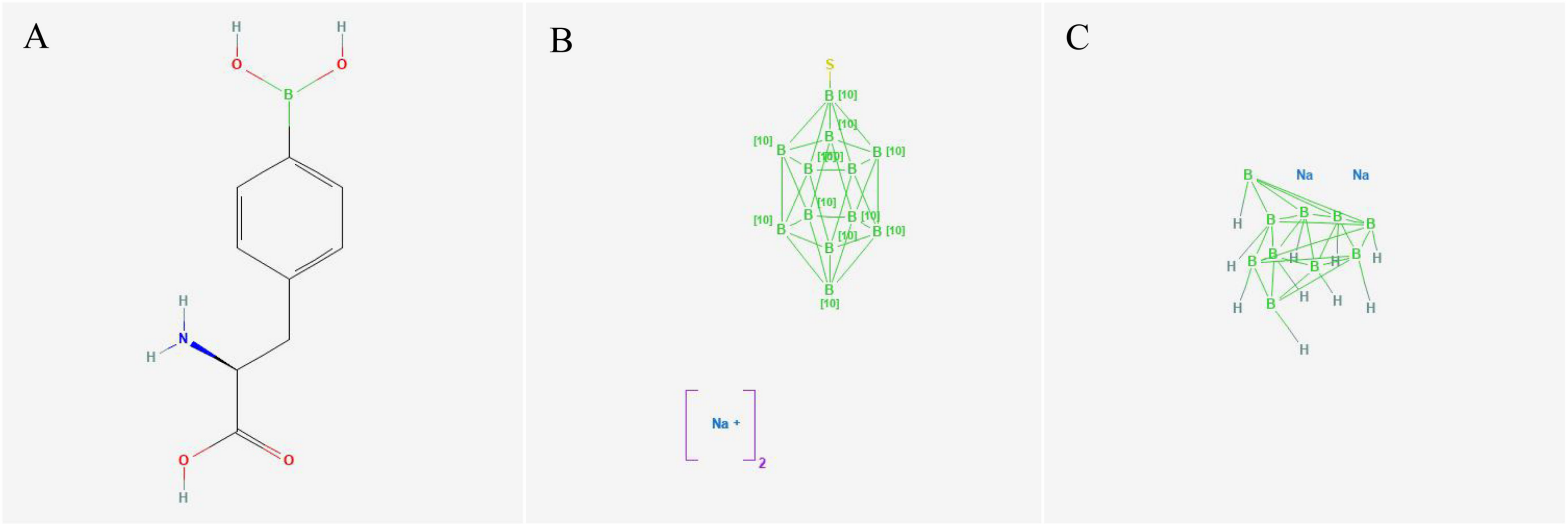
Structures of BPA, BSH, and GB-10. **(A)** BPA, https://pubchem.ncbi.nlm.nih.gov/compound/150315. **(B)** BSH, https://pubchem.ncbi.nlm.nih.gov/substance/198937381. **(C)** GB-10, https://pubchem.ncbi.nlm.nih.gov/substance/507727261.

In BNCT, a binary therapy approach, both the boron delivery agent and the neutron source play crucial roles. The evolution of boron delivery agents can be categorized into approximately three generations. The first generation of boron delivery agents, exemplified by boric acid, lack tumor targeting properties and result in serious adverse reaction, making them unsuitable for clinical applications (43, 44). Boronophenylalanine (BPA), sodium borocaptate (BSH), and decahydrodecaborate (GB-10) representing the second generation of boron delivery agents, are the only three drugs currently approved for clinical use (45). Their molecular structures are shown in **Figure 1**. BPA enters tumor cells via active transport through amino acid transporters. Its therapeutic efficacy is limited by low boron content, poor solubility, insufficient accumulation at the tumor site, and a short retention time. BSH and GB-10, which lack receptor-mediated tumor selectivity, enters tumor cells via passive transport. This results in a low tumor to blood (T/B) boron ratio and causes significant side effects during treatment. Furthermore, both BPA and BSH exhibit the problem of heterogeneous distribution within tumors in their clinical applications, significantly impacting the effectiveness of BNCT (46–48). Advancements in synthesis methods and a better understanding of biological characteristics have promoted the development of novel boron delivery agents. The third generation of boron delivery agents encompasses a range of low molecular weight compounds like amino acids, peptides, polyamines, nucleosides, carbohydrates, and porphyrins, as well as high molecular weight reagents such as liposomes, proteins, monoclonal antibodies, and nanoparticles (49, 50).

For boron agents to be considered high quality, they must fulfill specific criteria. These include the ability to target tumors effectively, be readily absorbed by tumor cells, and accumulate to a therapeutic concentration (20-30 micrograms of boron-10 per gram of tumor tissue) within the tumor, facilitating neutron irradiation therapy (49, 51). Moreover, they should have a minimal presence in normal tissues and be rapidly eliminated. Ideally, these agents should also distribute uniformly across all tumor cells and localize within or near the nucleus. If boron delivery agents can meet the above conditions, the efficacy of BNCT will be greatly improved (49, 51). In addition, high water solubility, good biocompatibility and low toxicity, excellent cellular permeability, chemical stability, and ease of synthesis are essential characteristics of high-quality boron delivery agents. Among these, enhanced targeting capability enables the precise and selective destruction of tumor cells while minimizing damage to normal tissues. Consequently, targeting has emerged as a central focus in the development of novel boron delivery agents.

Active and passive targeting represent two key mechanisms in drug delivery systems. Active targeting involves the specific interaction between drug carriers and molecular targets on the surface of target tissues or cells (such as receptors or antigens), thereby directing the drug to the intended site for precise therapeutic action. In contrast, passive targeting relies on the physicochemical properties of the drug or carrier (e.g., particle size, surface characteristics) and physiological processes (such as enhanced vascular permeability) to achieve drug accumulation in specific regions. Nanocarriers primarily exploit the enhanced permeability and retention (EPR) effect in tumor tissues to achieve natural accumulation at the tumor site. Several high-quality reviews have summarized the characteristics and applications of newly developed boron nanocarriers as well as the clinically approved agents BPA and BSH (35, 51–54). In this work, we surveyed the relevant literature from the past decade and provided a comprehensive summary of boron delivery agents with active tumor-targeting properties, including boron-based nanocarriers possessing such capabilities. Based on the location of the target, these agents can be categorized into those targeting the cell membrane, cytoplasm/organelles, nucleus, extracellular/secreted components, and the tumor microenvironment (**Supplementary Table 2**).

Common targets include the folate receptor, αvβ3 integrin, and epidermal growth factor receptor (EGFR). Folate-modified B_4_C nanoparticles exhibited favorable water solubility, stability, and low hemolytic and cytotoxic activity. *In vivo* experiments in tumor-bearing mice demonstrated significant tumor accumulation of these nanoparticles, with high tumor to muscle and tumor to brain ratios, indicating their suitability for BNCT applications (55). Compared with the random distribution of boron within cells, localization in the nucleus will reduce the required dose for treatment, thereby decreasing the neutron dose and side effects (38). DOX-CB@lipo-pDNA-iRGD was a multifunctional liposomal drug delivery system engineered for dual targeting of tumor cell surface receptors and the cell nucleus. The system employed the tumor-penetrating peptide iRGD to bind αvβ3 integrin receptors on tumor cells, enabling precise tumor tissue accumulation. Its nuclear-targeting and boron-carrying capabilities were conferred by the DOX-CB complex, comprising doxorubicin (DOX), a chemotherapeutic agent, and carborane (CB), a boron-rich compound (56). The nuclear translocation of DOX is attributed to its high-affinity binding to proteasomes in the cytoplasm. The DOX-proteasome complex enters the nucleus through nuclear pores in an ATP-dependent manner, a process that may be mediated by the nuclear localization signal (NLS) sequences present in the α subunits of the proteasome (57). By leveraging this intrinsic nuclear trafficking mechanism, the DOX-CB complex facilitates efficient boron delivery into the tumor cell nucleus, thereby enhancing the therapeutic efficacy of BNCT. Hybrid 1 selectively accumulated in glioma cells by targeting EGFR, with minimal impact on normal cells. In tumor-bearing mice, it demonstrated potent antitumor activity, significantly reducing tumor size and prolonging animal survival time (58).

A boron delivery agent that can be used for both diagnostic imaging and quantitative analysis of macroscopic ¹^0^B distribution, as well as serve as a boron carrier during the treatment process, can be termed a theranostic boron delivery agent (59). Its imaging capability provides the following advantages: verifying the targeting ability of the boron delivery agent, screening patients suitable for BNCT therapy, formulating personalized treatment plans, guiding the timing of treatment, and evaluating the therapeutic efficacy of BNCT. Over the past decade, a large number of boron delivery agents incorporating imaging capabilities have been designed and synthesized, which can be detected using techniques like PET, magnetic resonance imaging (MRI), fluorescence imaging, and other imaging modalities (**Supplementary Table 3**).

Chen et al. synthesized a novel boronated amino acid, trifluoroborate boronophenylalanine (BBPA), which exhibited high tumor uptake similar to BPA but with lower background uptake. This enabled [^18^F]BBPA-PET imaging to more clearly identify tumor boundaries, thereby improving the accuracy of tumor diagnosis. The diboron structure of BBPA endowed it with higher boron delivery efficiency. Compared to BPA, BBPA demonstrated a better BNCT therapeutic effect at the same dose (60). Sforzi et al. developed poly lactic-co-glycolic acid (PLGA) nanoparticles (PLGA-His) loaded with a theranostic agent, AT101, which contains an oligohistidine chain and a dual Gd/B chelate. By monitoring the distribution of Gd, the concentration of boron in the target tissue could be indirectly quantified, enabling image-guided BNCT. The PLGA nanoparticles could effectively deliver boron into mesothelioma cells, and after thermal neutron irradiation, both the survival rate and clonogenic ability of the tumor cells were significantly affected (61). Huang et al. synthesized a novel near-infrared fluorescent probe (PBA-BDP) by conjugating boron-10 to a BODIPY fluorescent dye modified with phenylboronic acid (PBA). Using *in vivo* fluorescence imaging, the biodistribution and tumor accumulation of PBA-BDP could be monitored in real-time and non-invasively, thereby guiding the optimal timing for BNCT treatment. Both *in vitro* and *in vivo* experiments showed that PBA-BDP could significantly inhibit tumor growth and induce apoptosis (62).

In light of the high costs and lengthy timelines associated with developing novel boron carriers, the clinical application of currently approved carriers is significantly hampered by several challenges, including poor water solubility, insufficient tumor accumulation, short retention times, and difficult clearance from normal tissues. Consequently, optimizing these existing agents represents a viable and strategic alternative.

The water solubility of 4-BPA is extremely low under neutral conditions (0.6–0.7 g/L), necessitating the addition of solubilizing sugars such as fructose or sorbitol. The use of solubilizing sugars may lead to certain side effects, including hypoglycemia, liver and kidney dysfunction, and hematuria. The solubility of the 3-isomer of BPA (3-BPA) was 10–100 times higher than that of the currently clinically used 4-isomer of BPA (4-BPA), and there was no significant difference in the uptake of 3-BPA and 4-BPA by melanoma-bearing mice (51, 63). Tyrosine kinase inhibitor-L-p-boronophenylalanine (TKI-BPA), a modification of BPA combined with targeted drugs, enhanced the tumor selectivity of the boron delivery agent. The uptake of TKI-BPA by gastric cancer cells and pancreatic cancer cells was significantly higher than that by normal cells, and the solubility of TKI-BPA was 6 times that of BPA (64). Poly(vinyl alcohol) (PVA) can form complexes with BPA, namely PVA-BPA. PVA-BPA entered cells via LAT1-mediated endocytosis and localized in endosomes/lysosomes, thereby bypassing the antiport mechanism of LAT1. Compared with the clinically used fructose-BPA complex, PVA-BPA exhibited higher accumulation and longer retention in tumors. Moreover, PVA-BPA was rapidly cleared from the blood and normal organs, reducing the risk of radiation damage to normal tissues. Furthermore, in a mouse model with colon cancer tumors, intravenous injection of PVA-BPA followed by neutron irradiation resulted in a marked decrease in tumor size (65). The complex composed of a fructose-modified poly(ethylene glycol)-poly(l-lysine) block copolymer and p-boronophenylalanine, termed PEG-P[Lys/Lys(fructose)]-BPA, not only achieved efficient enrichment and retention in tumors but also promoted renal clearance due to its moderate cationic properties. Under thermal neutron irradiation, PEG-P[Lys/Lys(fructose)]-BPA significantly inhibited tumor growth in mice (66).

AuNPs-BSH&PEG-cRGD was a cRGD-modified gold nanoparticle used as a carrier for BSH. In glioma cells, cRGD modification increased the boron content by 2.5 times through targeting the integrin receptor αvβ3. Moreover, six hours after administration, the boron concentration of AuNPs-BSH&PEG-cRGD in the glioma tumors of mice was significantly higher than that of BSH (17.98 μg/g vs. 0.45 μg/g) (67). Dodecaboranethiol (BSH)-containing kojic acid (KA-BSH) exhibited strong tumor-targeting capabilities, achieving significantly higher boron concentrations in glioma cells than both BPA and BSH (68). Block copolymer-boron cluster conjugate based on the clinically used sodium borocaptate (BSH) and poly(ethylene glycol)-b-poly(glutamic acid) copolymer, PEG-b-P(Glu-SS-BSH), enhanced not only the solubility but also the tumor-targeting properties of BSH, promoting its even distribution within colon and pancreatic cancer tissues. Additionally, in a mouse model with subcutaneously implanted colon tumors, the combination of PEG-b-P(Glu-SS-BSH) and thermal neutron irradiation effectively suppressed tumor growth (69).

Masunaga et al. investigated the potential of liposomes with varying surface modifications—including bare liposomes, polyethylene glycol (PEG)-modified liposomes, and transferrin-pendant-type PEG (TF-PEG) liposomes—as carriers for GB-10 in delivering boron to tumor cells for BNCT. The results demonstrated that encapsulation of GB-10 within these liposomal formulations significantly enhanced boron accumulation in tumors and markedly improved the tumor cell-killing effect following neutron irradiation. Among the formulations evaluated, TF-PEG liposomes demonstrated the highest therapeutic efficacy (70).

The method of administering the boron delivery agent may influence the effectiveness of BNCT. In the F98 rat glioma model, numerous studies explored how the method of drug delivery impacted the concentration of boron in tumors and the survival duration of the rats. The findings indicated that, compared with those receiving intravenous treatment, rats treated with intracarotid artery administration, coupled with the disruption of the blood-brain barrier, presented a nearly fourfold higher concentration of boron in tumors. This group also experienced a notable extension in MST (71–77). Therefore, exploring new drug delivery methods can help to improve the therapeutic effectiveness of BNCT. Delivery methods such as focused ultrasound, electroporation, cerebrospinal fluid (CSF) circulation, and microneedles have shown promising prospects (78–85). High-intensity focused ultrasound can increase the uptake of 18F-BPA by oral squamous cell carcinoma tumors transplanted into nude mice, without increasing the uptake of 18F-BPA in normal tissues (85). The blood-brain barrier hinders the delivery of drugs to brain tumors, thereby affecting the effectiveness of treatments. Kusaka et al. attempted to increase the efficacy of BNCT by delivering BPA to gliomas through the CSF circulation. Both CSF administration and intravenous methods of drug delivery demonstrated significant therapeutic benefits. Notably, rats treated with CSF administration presented reduced accumulation of BPA in healthy tissues while maintaining their overall well-being (79). PVA/F-BPA microneedles are a transdermal drug delivery system in which F-BPA is loaded into the tips of PVA microneedles. Boron was effectively delivered to mouse melanoma via PVA/F-BPA microneedles, achieving a remarkable T/N ratio of 93.16. This BNCT treatment significantly suppressed melanoma growth and notably improved the survival rate of the mice (80). Interestingly, Khan et al. compared the effects of direct intratumoral injection versus systemic delivery of boron-rich liposomes on the outcomes of BNCT for breast cancer. The results showed that systemic delivery of boron-rich liposomes combined with neutron irradiation could alter the phenotype of peripheral blood mononuclear cells, shifting them toward an anti-tumor phenotype. This was characterized by increased expression of the cytokine IL-12 and decreased expression of IL-10, thereby inhibiting tumor growth (86). Therefore, when developing therapeutic agents, it is essential to determine the optimal delivery method.

### Neutron source

In addition to the boron delivery agent, the neutron source plays a vital role as another essential component in BNCT therapy. Before 2015, nuclear reactors were the sole source of neutrons for clinical BNCT, and the output of these reactors was often difficult to control. Additionally, the initial thermal neutron beams used in BNCT had their peak flux at a depth of 2–3 centimeters below the skin, which limited the effectiveness of the treatment (38). The introduction of epithermal neutron beams in the 1990s met the treatment requirements for deep-seated tumors (87). Since 2015, accelerator-based neutron sources that can be installed in hospitals have been utilized for clinical treatment (88). In contrast to nuclear reactors, accelerator-based neutron sources have several strengths. For instance, they are smaller in size, have lower maintenance costs, and are more easily adjusted to meet the specific parameters needed for therapeutic neutron beams (38). Before any boron delivery agents can be considered for clinical trials in BNCT treatment, they must undergo rigorous testing in both cellular and animal models. A significant hurdle worldwide is the scarcity of neutron sources available for these essential experimental studies (88).

### Treatment planning system

The treatment planning system is a key component of the BNCT treatment process, directly affecting the outcome of the therapy and the overall health and survival of the patient. The treatment planning system ensures the precision of BNCT through several key steps. Initially, it utilizes high-resolution imaging to map the tumor’s location, size, shape, and the status of surrounding healthy tissues. Next, it evaluates the distribution of the boron delivery agent within the patient’s body. It then simulates the neutron beams path and interaction with tissues. The system also calculates the neutron dose distribution in both tumor and normal tissues, optimizes the treatment plan with advanced algorithms, and verifies its accuracy via dedicated tools before treatment. During therapy, it continuously monitors the performance of the treatment equipment. Hence, refining the software and algorithms within the treatment planning system is crucial for enhancing the outcomes of BNCT.

During neutron irradiation, in addition to boron, interactions between neutrons and nitrogen and hydrogen atoms can lead to secondary radiation, which may harm surrounding healthy tissues. Researches indicated that higher radiation doses were linked to an increased incidence of side effects and reduced PFS and OS in patients receiving BNCT (89, 90). Thus, it is essential to precisely estimate the neutron irradiation dose needed for therapy. A dose that is too low might not fully eliminate tumor cells, whereas a dose that is too high could result in side effects and reduce survival duration. The accurate calculation of the neutron dose is inseparable from the exact assessment of the boron concentration. The synthesis of ^18^F-BPA drugs and the development of ^18^F-BPA-PET technology have simplified the monitoring of boron concentration (91, 92). This approach allows for a noninvasive assessment of boron levels in tumor tissues prior to BNCT, aiding in the selection of patients who are likely to benefit from the treatment and enabling a more accurate determination of the necessary neutron irradiation dose for therapy (93).

The distribution of boron in tumors is nonhomogeneous. However, most treatment planning systems estimate neutron doses under the assumption of uniform boron distribution within the tumor. Studies by Teng et al. revealed that this assumption might result in an overestimation of the minimum dose rate D_min_ and 80% dose rate D_80_, leading to inadequate irradiation. This insufficiency might increase the risk of tumor recurrence and progression (94). Sato et al. proposed a new model for estimating the biological effectiveness of BNCT, which considered the heterogeneity of the boron distribution both intracellularly and intercellularly. The results suggested that considering this variability might lead to a more precise evaluation of the therapeutic impact of BNCT (95). Consequently, optimizing the software and algorithms used in treatment systems will be beneficial for enhancing the outcomes of BNCT.

### Tumor heterogeneity

Tumor tissue is composed of tumor cells and the surrounding environment that supports their survival. This environment consists of various cellular elements such as fibroblasts, endothelial cells, and immune cells, as well as noncellular components like the ECM, blood and lymphatic vessels, and a range of biologically active molecules. Variations exist both among the tumor cells themselves and within the microenvironment that sustains them. This variability can be attributed to several factors, including genetic and phenotypic variations among tumor cells, the diverse array of cellular elements within the tumor tissue, and the complex microenvironment surrounding these cells, collectively known as tumor heterogeneity (96).

Tumor heterogeneity refers to differences in the molecular characteristics and phenotypes (such as growth rate, apoptosis, response to drugs, etc.) of tumor cells. When this diversity is observed across different patients with tumors in the same organ, it is known as intertumor heterogeneity. When variations exist in a single patient, either across different areas of the tumor or between the original tumor and any recurrences in the same area or distant metastases, they are referred to as intratumor heterogeneity, namely spatial heterogeneity and temporal heterogeneity (97, 98). Interumor heterogeneity might highlight the variations at the level of the entire patient population, offering insight into why patients with the same tumor type might respond differently to the same treatment. Intratumor heterogeneity might potentially explain cases where a patient shows initial improvement with treatment but later faces a relapse of the disease (99).

Tumor heterogeneity might lead to uneven distribution of boron in the tumor, which in turn affected the therapeutic effect of BNCT (46, 48, 95, 100, 101). The study by Tamura et al. disclosed how tumor diversity interacted with BNCT outcomes. A meningioma patient initially had a boron concentration T/N ratio of 5.0, which decreased to 1.9 upon recurrence, indicating inherent diversity in the original tumor. This diversity meant that some parts of the tumor absorbed boron more effectively than others did. As a result, after BNCT, areas with higher boron absorption were significantly damaged, whereas cells in regions with lower absorption survived, leading to a relapse. This diversity influenced the effectiveness of BNCT. The reduced T/N ratio in the recurrent tumors suggested that BNCT modified the state of tumor heterogeneity (102). Some studies have shown the impact of tumor heterogeneity on boron distribution, proposing methods to enhance boron distribution and strategies to manage instances of uneven boron spread. For example, combining boron carriers with different uptake mechanisms helps achieve more uniform tumor targeting. Heber et al. used three different drug regimens to examine how boron was distributed in a hamster cheek pouch oral cancer model. They found that the combination of BPA and GB-10 significantly improved the uniformity of boron distribution compared with the use of BPA alone (103). Subsequent research demonstrated that this combination, followed by BNCT, induced a rapid and long-lasting response (19).

The presence of quiescent cells is also a manifestation of tumor heterogeneity. When faced with adverse microenvironments such as hypoxia and nutrient deprivation, tumor cells could exit the cell cycle and enter a quiescent state. These quiescent cells disrupt the even spread of boron, which could reduce the effectiveness of BNCT (104). For example, in a subcutaneous chicken cell virus-induced tumor (SCCVII) model, the inability of quiescent cells to accumulate BPA might lead to the recurrence of cancer after BNCT (25). Hypoxic cells are more common in quiescent cells than in proliferating ones. Enhancing blood flow could help promote the return of quiescent cells to the proliferative state, reducing tumor heterogeneity. Studies indicated that the use of BNCT in conjunction with drugs targeting hypoxic cells, such as tirapazamine, or mild temperature hyperthermia that enhances blood flow, could effectively control tumor growth (105, 106).

Tumor cells exhibit differences in boron uptake and response to BNCT not only between quiescence and proliferation but also across various stages of the cell cycle. Cells such as V79, SCCVII, and C6 absorbed boron more efficiently during the G2/M phase than in the G0/G1 phase. Moreover, BPA showed a more pronounced cell cycle dependency compared to BSH (107). Similarly, in the human cervical cancer cell line HeLa, the uptake of BPA varied with the cell cycle, with significantly higher concentrations in the S/G2/M phase than in the G1/S phase. The disparity in BPA levels results in different responses to BNCT, with G1/S phase cells showing lower sensitivity to radiation compared to S/G2/M phase cells. Treatment with PVA-BPA increased the boron levels to approximately twice that of cells treated with BPA alone, without a significant difference between G1/S and S/G2/M phase cells. PVA-BPA enhanced boron uptake, addressed the uneven distribution caused by cell cycle variations, and improved the effectiveness of BNCT (108). These findings implied that combining BNCT with cell cycle-specific anticancer drugs could enhance the therapeutic effect of BNCT.

The diversity within the tumor microenvironment also affects the effectiveness of BNCT. Yu et al. used a 3D tumor spheroid platform to evaluate the impact of BNCT on pancreatic tumor spheroids of different diameters under various microenvironmental conditions. They reported that larger spheroids had lower sensitivity to BNCT. Further analysis revealed that hypoxia and fibrosis might reduce the efficacy of BNCT in pancreatic cancer treatment by influencing the HIF1-α signaling pathway and β-catenin nuclear translocation (109).

Overall, developing new boron delivery agents to increase tumor uptake or combining other treatments to improve boron distribution within tumors can enhance the effectiveness of BNCT. The impact of the microenvironment on BNCT demands careful consideration. Various factors influence drug delivery from the blood to the cells, including vessel patency, vessel permeability and tissue permeation. Combining drugs that target tumor vasculature or fibrotic stroma could enhance drug delivery, such as antiangiogenic therapies that induce vascular normalization and methods to reduce tumor fibrosis and solid stress (110–115).

## Challenges faced by BNCT

Tumor targeting, effective accumulation and retention in tumor tissue, minimal or no presence in normal tissues, rapid clearance from normal tissues, low toxicity, and high solubility are all essential characteristics for an ideal boron delivery agent. All these properties are aimed at enhancing the therapeutic efficacy of BNCT and protecting normal tissues from damage. In recent years, a large number of boron delivery agents have been developed to meet these requirements. Selective accumulation of boron in tumor tissue and the protection of normal tissues can be achieved by actively targeting receptors that are highly expressed on tumor cells, such as folate receptors and integrin receptors, or by leveraging the EPR effect of tumor tissue. The development of theranostic boron delivery agents is expected to enable BNCT treatment guided by imaging. Nanotechnology has shown unique advantages in the development of boron delivery agents due to its high drug-loading capacity, ease of functional modification, and multi-modal imaging capabilities. However, it is crucial to address the potential toxicity of nanoparticles, such as their accumulation in the liver, spleen, lungs, and kidneys, and the induction of oxidative stress (53).

Although a large number of boron delivery agents have been developed, most remain at the cellular and animal experiment stages. All agents can only proceed to subsequent production and application after being evaluated in clinical trials. The insufficiency of neutron source is a major factor limiting this progress. Solving this problem is inseparable from the support of national governments. Current clinical studies have shown that BNCT has promising efficacy in various cancers. Notably, these studies exhibited considerable heterogeneity regarding the criteria for subject inclusion and the endpoints used to assess BNCT efficacy. In the future, it is imperative to conduct well-designed randomized clinical trials to confirm the effectiveness and safety of BNCT and to establish standardized treatment protocols to provide guidance for clinical decision-making.

Within a single tumor, both quiescent cells and proliferating cells coexist, with cells at various stages of the cell cycle present simultaneously. The tumor microenvironment can vary from region to region, exhibiting characteristics such as adequate blood flow, hypoxia, or inflammation. This tumor heterogeneity would impact the distribution of boron in tumors. The evaluation of the boron concentration and calculation of the neutron dose are critical steps in the BNCT treatment process. Accurate assessment of the boron concentration within the tumor is essential for calculating the precise neutron dose needed for effective irradiation. Inadequate irradiation can result in tumor recurrence and progression, whereas excessive dosage may introduce side effects and decrease the patient survival. The ^18^F-BPA-PET technology has simplified the process of monitoring boron concentrations (92). Models that account for the cellular heterogeneity of boron distribution could provide more accurate estimates of the therapeutic efficacy of BNCT (95). Consequently, optimizing the software and algorithms used in BNCT treatment planning systems is crucial for improving treatment outcomes.

On top of affecting the uniform distribution of boron, tumor heterogeneity may further influence the response of tumor cells to neutron irradiation, and this could be associated with disease recurrence. Scientists have implemented several strategies to reduce the undesirable effects of tumor heterogeneity. Reviving quiescent cells into a proliferative state and addressing the uneven boron distribution due to differences in the cell cycle have both shown potential in enhancing the outcomes of BNCT. Additionally, targeting the tumor microenvironment to optimize drug delivery is also a promising research direction. In addition, the use of additional X-ray therapy (XRT) can compensate for the inadequate neutron irradiation doses resulting from uneven boron distribution within tumor tissues (116). Extra XRT has significantly prolonged the survival of patients with glioma (117). Therefore, to improve the therapeutic efficacy of BNCT, the existence of tumor heterogeneity must be considered in all aspects, including the development of boron delivery agents, the calculation of neutron dosimetry, and the decision on whether to combine it with other therapeutic modalities.

In addition, BNCT faces several economic and logistical challenges, such as the research and development costs for boron delivery agents, the capital investment in building specialized BNCT facilities, the training of medical personnel, as well as the treatment expenses and reimbursement models associated with patient care.

## Discussion

The development of novel, high-quality, multifunctional boron delivery agents is crucial for advancing BNCT into clinical practice. Optimizing the carriers already approved for clinical trials is a promising and feasible approach to developing new agents. Furthermore, increasing tumor boron uptake, utilizing radiosensitizers, and combining BNCT with other treatment modalities can significantly enhance therapeutic efficacy.

BPA is a boron-containing phenylalanine derivative that is selectively transported into tumor cells via amino acid transporters. Therefore, upregulating the expression of amino acid transporters can enhance tumor uptake of BPA, thereby improving the therapeutic efficacy of BNCT. Sodium butyrate could upregulate the expression of LAT protein, a transporter for BPA, leading to increased absorption of BPA in thyroid cancer cells (118). The amino acid 5-aminolevulinic acid potentially enhanced the absorption of BPA in glioma stem cells by elevating the expression of amino acid transporters (119). In glioblastoma cells in which LAT1 was overexpressed, the absorption of BPA was significantly increased, ranging from 1.5 to 5.0 times higher than that in the control cells. Moreover, the level of BPA uptake was closely related to the sensitivity of cancer cells to radiation therapy (120).

The combination of radiosensitizers with BNCT has been shown to significantly enhance therapeutic outcomes. Valproic acid (VPA), a well-established histone deacetylase inhibitor, markedly reduced the viability of melanoma cells when administered in conjunction with BNCT. The underlying molecular mechanisms involved the following sequential events: First, VPA potentiated DNA damage by modulating chromatin architecture, thereby amplifying BNCT-induced DNA double-strand breaks. Second, VPA abrogated the G2/M cell cycle checkpoint, forcing prematurely damaged cells into mitotic progression and thereby eliminating the critical time window required for DNA damage repair. Third, VPA suppressed key DNA repair pathways by downregulating the expression of essential proteins including RAD51 recombinase (Rad51), Ku autoantigen 70 kDa (Ku70), and Ku autoantigen 80 kDa (Ku80), which are crucial for homologous recombination and non-homologous end joining repair, respectively. Collectively, these coordinated actions impaired the DNA damage response machinery, rendering melanoma cells unable to recover from genotoxic stress, which ultimately culminated in apoptotic cell death and significantly improved the therapeutic efficacy of BNCT (86).

Similar to other treatment methods, BNCT is also unable to prevent tumor recurrence and progression. Researchers are attempting to combine BNCT with other treatment modalities, such as chemotherapy, radiotherapy, immunotherapy, chemodynamic therapy, and photothermal therapy, to better control the disease and reduce the side effects linked to dose escalation in single-modality treatments. Among these, the combination with immunotherapy is particularly noteworthy (121–126). While killing tumor cells, BNCT can protect nearby normal cells, including immune cells, from damage. BNCT treatment reduces the tumor burden, making it easier for the immune system to recognize and eliminate residual cancer cells. Furthermore, the direct biological effect of BNCT is the induction of DNA double-strand breaks. The DNA damage caused by BNCT is more numerous and complex, making it difficult to repair, which ultimately leads to cell death. When DNA is damaged, cell division often results in the formation of micronuclei (127). The nuclear membranes of these micronuclei are fragile and prone to rupture, exposing DNA in the cytoplasm and thereby activating the innate immune cyclic GMP-AMP synthase-stimulator of interferon genes (cGAS-STING) signaling pathway (128). As tumor cells undergo death following BNCT treatment, they release a series of signaling molecules, such as surface-exposed calreticulin (CRT), secreted ATP, and extracellular HMGB1, which trigger adaptive immune response; this mode of cell death is known as immunogenic cell death (ICD) (129, 130). Moreover, the abscopal effect induced by BNCT treatment may be attributed to the systemic immune response activated after the treatment (123, 131, 132). Therefore, the combination of BNCT and immunotherapy holds great promise for achieving a more potent synergistic effect.

Although the combination of BNCT and immunotherapy has a strong theoretical basis, an issue that cannot be overlooked is that BNCT can induce immunosuppression through certain mechanisms. Studies have shown that radiotherapy can upregulate the expression of programmed death-ligand 1 (PD-L1) in tumor cells (133). As a form of radiotherapy, BNCT could also elevate the levels of PD-L1 in tumor cells (134). By highly expressing PD-L1, tumor cells bind to programmed death-1 (PD-1) on the surface of T cells, inhibiting their activity and thus evading immune recognition and clearance. Therefore, researchers have attempted to combine the use of PD-1 or PD-L1 antibodies to improve therapeutic outcomes. In a mouse model of advanced melanoma resistant to both radiotherapy and immunotherapy, the combination of BNCT and anti-PD-1 immunotherapy demonstrated a significant inhibitory effect on tumor growth at both the BNCT-treated site and the shielded site (123). Compared to BNCT alone, the combination of BNCT and anti-PD-L1 immunotherapy significantly prolonged the tumor growth delay time in melanoma mice. Immunohistochemical staining results revealed increased T cell infiltration in the tumor, indicating an enhanced immune response (124).

PD-L1 on the tumor cell membrane can act as a “molecular shield” to suppress the immune system, while intracellular PD-L1, functioning as an RNA-binding protein, can bind and stabilize mRNAs related to DNA damage repair (133, 134), thereby limiting the DNA damage effects induced by BNCT (135, 136). Even when PD-L1 on the cancer cell membrane is inhibited by a PD-L1 antibody, a continuous cycle transports PD-L1 from inside the cell to the surface to replenish it (137). In other words, antibody drugs cannot inhibit the expression of intracellular PD-L1. To address this, a novel boron delivery agent was synthesized, consisting of nanoparticles self-assembled from a 10B-containing copolymer and PD-L1 siRNA. The 10B/siPD-L1 nanoparticle therapy combined with BNCT precisely killed tumor cells through a dual mechanism of enhancing DNA damage and inhibiting immune checkpoints, effectively activating an anti-tumor immune response and inhibiting distant and metastatic tumors (134).

MDSCs are a heterogeneous group of myeloid cells that weaken the body’s ability to clear tumors by suppressing NK cell activity, inhibiting T cell proliferation, and polarizing macrophages via the activation of the colony-stimulating factor-1/colony-stimulating factor-1 receptor (CSF-1/CSF-1R) pathway. In a mouse model of 4-Nitroquinoline N-oxide (4-NQO)-induced head and neck squamous cell carcinoma, BNCT led to a transient decrease in M-MDSCs in the peripheral blood and tumors, followed by a continuous increase. MDSCs express CSF-1R. The use of the CSF-1R inhibitor PLX3397 effectively suppressed the BNCT-induced circulating M-MDSCs, as well as tumor-infiltrating M-MDSCs and tumor-associated macrophages, leading to an increase in CD8+ T cells. The combination of BNCT and PLX3397 significantly prolonged the survival time of the mice (138).

Antigens released by macrophages after engulfing tumor cells are further processed and presented by dendritic cells (DCs), enhancing T cell activation. Insufficient phagocytic function of macrophages and inadequate antigen-presenting capacity of dendritic cells can both impair the immune system’s ability to clear tumor cells. Cancer cells, particularly cancer stem cells, often overexpress cluster of differentiation 47 (CD47). When CD47 binds to signal regulatory protein alpha (SIRPα) on the surface of macrophages, it triggers a “don’t eat me” signal, thereby inhibiting phagocytosis (139, 140). The multifunctional nanolipid drug delivery system DOX-CB@lipo-pDNA-iRGD activated macrophage-mediated phagocytosis and promoted adaptive immunity through knockout of the CD47 gene. The combination of BNCT and CD47 blockade significantly improved the survival rate of tumor-bearing mice (56).

Researchers co-cultured 10B-doped carbon dots (10B-CDs) with RM-1 prostate cancer cells and encapsulated the 10B-CDs in freeze-dried cancer cells (L-RM1@10B-CDs) using a liquid nitrogen freeze-drying technique. L-RM1@10B-CDs served both as a source of tumor cell antigens and a delivery vehicle for the 10B-CDs. *In vitro* studies showed that L-RM1@10B-CDs could be efficiently taken up by antigen-presenting cells (APCs) and promoted APC maturation and activation of the immune response. *In vivo* studies revealed that BNCT mediated by L-RM1@10B-CDs shifted the immune status of the tumor tissue from “cold” to “hot,” significantly inhibiting the growth of prostate cancer in mice (141).

The research of Chen et al. also found that the immune response induced by BNCT is limited by low antigen presentation efficiency and insufficient DCs maturation. Therefore, they developed a boron nitride nanosystem loaded with the immunostimulant imiquimod (R837). After neutron irradiation, tumor cells that had internalized the boron-containing formulation acquired the properties of BNCT-shocked tumor cells (BTCs) loaded with R837. R837-loaded BTCs were more efficiently taken up by DCs, promoting DCs maturation and the secretion of pro-inflammatory cytokines. After subcutaneous immunization of mice with BTCs, infiltration of CD4+ and CD8+ T cells in the tumor tissue significantly increased, while immunosuppressive regulatory T cells decreased. These BTCs can co-deliver tumor antigens and an immunoadjuvant to lymph nodes, activating a potent anti-tumor immune response. This strategy not only effectively inhibited primary tumors and distant metastases but also induced long-term immune memory (142).

Tumors create an immunosuppressive environment that allows them to evade destruction by the immune system. Combination therapy with immunoadjuvants is one of the effective methods to overcome immunosuppression and improve the immune response (143). The combination of BNCT and bacillus calmette-guerin (BCG) for treating colon cancer induced local, regional, and abscopal effects (131). Researchers have designed and synthesized boron capsules loaded with the immunoadjuvant imiquimod. Neutron irradiation could accelerate the drug release from these boron capsules. The combined treatment of BNCT with the boron capsules significantly inhibited the growth of both primary and distant tumors and prolonged the survival time of the mice. Mechanistic studies suggested that BNCT increased the number of tumor-infiltrating immune cells and converted immunosuppressive tumors into immunogenic ones. Furthermore, the sustained release of imiquimod promoted the polarization of macrophages, further enhancing the anti-tumor immune response (144).

As mentioned above, developing novel, high-quality boron delivery agents and modifying the clinically approved boron carriers are crucial. Concurrently, exploring strategies to enhance boron uptake in tumor cells through gene therapy, combining BNCT with radiosensitizers, and integrating it with emerging treatment modalities such as immunotherapy are promising approaches to improve the therapeutic efficacy of BNCT.

To advance BNCT from a cutting-edge technology to a standard clinical practice, a significant amount of work and a long period of development are still required. First, the development of novel, multifunctional boron delivery agents with higher tumor targeting specificity and lower toxicity, the optimization of neutron source technology, and the construction of intelligent treatment planning systems are key to promoting the technological iteration and advancement of BNCT. Second, to accelerate its clinical translation and widespread adoption, government bodies and relevant agencies should increase funding to incentivize basic research and the development of critical equipment. It is also essential to establish dedicated approval pathways and scientific evaluation standards for BNCT to streamline processes and improve efficiency. Furthermore, exploring the feasibility of including BNCT in medical insurance systems should be actively pursued to alleviate the financial burden on patients, thereby enhancing its clinical accessibility. Third, a unified and standardized framework for clinical operations and evaluation must be established. This includes formulating authoritative clinical guidelines to clearly define indications, accurately identify patient populations who would benefit, standardize treatment protocols, and establish criteria for efficacy evaluation. Concurrently, systematic long-term follow-up is necessary to assess long-term efficacy and safety, providing robust data to support the continuous optimization of clinical practice. In addition, cultivating a professional workforce proficient in the core technologies of BNCT is an indispensable component of achieving standardized treatment. Finally, the advancement of BNCT is highly dependent on close collaboration across multiple fields, including nuclear medicine, oncology, radiation physics, nuclear engineering, and even the biopharmaceutical industry. By promoting the industrialization of BNCT-related drugs and equipment and accelerating the translation of research findings into clinical applications, resources can be effectively integrated to overcome technical bottlenecks, ultimately realizing the widespread application of BNCT.

In summary, the numerous challenges currently facing BNCT can only be systematically overcome through the organic integration of technological innovation, policy support, clinical standardization, and cross-disciplinary collaboration to create a synergistic effect. This will enable it to truly become a powerful tool in the field of cancer therapy, bringing significant benefits to a vast number of patients.

## References

[1] Bray F Laversanne M Sung H Ferlay J Siegel RL Soerjomataram I. Global cancer statistics 2022: GLOBOCAN estimates of incidence and mortality worldwide for 36 cancers in 185 countries. CA Cancer J Clin. (2024) 74:229–63. doi: 10.3322/caac.21834, PMID: 38572751

[2] Wu Z Xia F Lin R. Global burden of cancer and associated risk factors in 204 countries and territories, 1980-2021: a systematic analysis for the GBD 2021. J Hematol Oncol. (2024) 17:119. doi: 10.1186/s13045-024-01640-8, PMID: 39614359 PMC11607901

[3] Taylor. HJ Goldhaber M. Detection of nuclear disintegration in a photographic emulsion. nature. (1935) 135:341–1. doi: 10.1038/135341a0

[4] Locher G. Biological effects and therapeutic possibilities of neutrons. Am J Roentgenol Radium Ther Nucl Med. (1936) 36:1–13.

[5] Zhou T Igawa K Kasai T Sadahira T Wang W Watanabe T. The current status and novel advances of boron neutron capture therapy clinical trials. Am J Cancer Res. (2024) 14:429–47. doi: 10.62347/hbbe6868, PMID: 38455422 PMC10915318

[6] Suzuki M Kato I Aihara T Hiratsuka J Yoshimura K Niimi M. Boron neutron capture therapy outcomes for advanced or recurrent head and neck cancer. J Radiat Res. (2014) 55:146–53. doi: 10.1093/jrr/rrt098, PMID: 23955053 PMC3885131

[7] Kankaanranta L Seppälä T Koivunoro H Saarilahti K Atula T Collan J. Boron neutron capture therapy in the treatment of locally recurred head-and-neck cancer: final analysis of a phase I/II trial. Int J Radiat Oncol Biol Phys. (2012) 82:e67–75. doi: 10.1016/j.ijrobp.2010.09.057, PMID: 21300462

[8] Capala J Stenstam BH Sköld K Munck af Rosenschöld P Giusti V Persson C. Boron neutron capture therapy for glioblastoma multiforme: clinical studies in Sweden. J Neurooncol. (2003) 62:135–44. doi: 10.1007/bf02699940, PMID: 12749709

[9] Yamamoto T Nakai K Kageji T Kumada H Endo K Matsuda M. Boron neutron capture therapy for newly diagnosed glioblastoma. Radiother Oncol. (2009) 91:80–4. doi: 10.1016/j.radonc.2009.02.009, PMID: 19285355

[10] Kageji T Mizobuchi Y Nagahiro S Nakagawa Y Kumada H. Long-survivors of glioblatoma treated with boron neutron capture therapy (BNCT). Appl Radiat Isot. (2011) 69:1800–2. doi: 10.1016/j.apradiso.2011.03.021, PMID: 21463946

[11] Yamamoto T Matsumura A Nakai K Shibata Y Endo K Sakurai F. Current clinical results of the Tsukuba BNCT trial. Appl Radiat Isot. (2004) 61:1089–93. doi: 10.1016/j.apradiso.2004.05.010, PMID: 15308197

[12] Miyatake S Kawabata S Yokoyama K Kuroiwa T Michiue H Sakurai Y. Survival benefit of Boron neutron capture therapy for recurrent Malignant gliomas. J Neurooncol. (2009) 91:199–206. doi: 10.1007/s11060-008-9699-x, PMID: 18813875

[13] Kawabata S Hiramatsu R Kuroiwa T Ono K Miyatake S. Boron neutron capture therapy for recurrent high-grade meningiomas. J Neurosurg. (2013) 119:837–44. doi: 10.3171/2013.5.jns122204, PMID: 23808536

[14] Takai S Wanibuchi M Kawabata S Takeuchi K Sakurai Y Suzuki M. Reactor-based boron neutron capture therapy for 44 cases of recurrent and refractory high-grade meningiomas with long-term follow-up. Neuro Oncol. (2022) 24:90–8. doi: 10.1093/neuonc/noab108, PMID: 33984146 PMC8730746

[15] Mishima Y Honda C Ichihashi M Obara H Hiratsuka J Fukuda H. Treatment of Malignant melanoma by single thermal neutron capture therapy with melanoma-seeking 10B-compound. Lancet. (1989) 2:388–9. doi: 10.1016/s0140-6736(89)90567-9, PMID: 2569577

[16] Hiratsuka J Kamitani N Tanaka R Tokiya R Yoden E Sakurai Y. Long-term outcome of cutaneous melanoma patients treated with boron neutron capture therapy (BNCT). J Radiat Res. (2020) 61:945–51. doi: 10.1093/jrr/rraa068, PMID: 32990318 PMC7674695

[17] Debatin KM Krammer PH. Death receptors in chemotherapy and cancer. Oncogene. (2004) 23:2950–66. doi: 10.1038/sj.onc.1207558, PMID: 15077156

[18] Okada H Mak TW. Pathways of apoptotic and non-apoptotic death in tumor cells. Nat Rev Cancer. (2004) 4:592–603. doi: 10.1038/nrc1412, PMID: 15286739

[19] Aromando RF Heber EM Trivillin VA Nigg DW Schwint AE Itoiz ME. Insight into the mechanisms underlying tumor response to boron neutron capture therapy in the hamster cheek pouch oral cancer model. J Oral Pathol Med. (2009) 38:448–54. doi: 10.1111/j.1600-0714.2008.00720.x, PMID: 19141057

[20] Rodriguez C Carpano M Curotto P Thorp S Casal M Juvenal G. *In vitro* studies of DNA damage and repair mechanisms induced by BNCT in a poorly differentiated thyroid carcinoma cell line. Radiat Environ Biophys. (2018) 57:143–52. doi: 10.1007/s00411-017-0729-y, PMID: 29453554

[21] Faião-Flores F Coelho PR Arruda-Neto JD Maria-Engler SS Maria DA. Cell cycle arrest, extracellular matrix changes and intrinsic apoptosis in human melanoma cells are induced by Boron Neutron Capture Therapy. Toxicol In Vitro. (2013) 27:1196–204. doi: 10.1016/j.tiv.2013.02.006, PMID: 23462526

[22] Sato A Itoh T Imamichi S Kikuhara S Fujimori H Hirai T. Proteomic analysis of cellular response induced by boron neutron capture reaction in human squamous cell carcinoma SAS cells. Appl Radiat Isot. (2015) 106:213–9. doi: 10.1016/j.apradiso.2015.08.001, PMID: 26302661

[23] Perico D Tong Y Chen L Imamichi S Sanada Y Ishiai M. Proteomic characterization of SAS cell-derived extracellular vesicles in relation to both BPA and neutron irradiation doses. Cells. (2023) 12:1562. doi: 10.3390/cells12121562, PMID: 37371031 PMC10296566

[24] Kinashi Y Sakurai Y Masunaga S Suzuki M Nagata K Ono K. Evaluation of micronucleus induction in lymphocytes of patients following boron-neutron-capture-therapy: a comparison with thyroid cancer patients treated with radioiodine. J Radiat Res. (2007) 48:197–204. doi: 10.1269/jrr.06086, PMID: 17485918

[25] Ono K Masunaga SI Kinashi Y Takagaki M Akaboshi M Kobayashi T. Radiobiological evidence suggesting heterogeneous microdistribution of boron compounds in tumors: its relation to quiescent cell population and tumor cure in neutron capture therapy. Int J Radiat Oncol Biol Phys. (1996) 34:1081–6. doi: 10.1016/0360-3016(95)02180-9, PMID: 8600091

[26] Kondo N Michiue H Sakurai Y Tanaka H Nakagawa Y Watanabe T. Detection of γH2AX foci in mouse normal brain and brain tumor after boron neutron capture therapy. Rep Pract Oncol Radiother. (2016) 21:108–12. doi: 10.1016/j.rpor.2014.10.005, PMID: 26933392 PMC4747666

[27] Masutani M Baiseitov D Itoh T Hirai T Berikkhanova K Murakami Y. Histological and biochemical analysis of DNA damage after BNCT in rat model. Appl Radiat Isot. (2014) 88:104–8. doi: 10.1016/j.apradiso.2014.03.003, PMID: 24690552

[28] Scaffidi P Misteli T Bianchi ME. Release of chromatin protein HMGB1 by necrotic cells triggers inflammation. Nature. (2002) 418:191–5. doi: 10.1038/nature00858, PMID: 12110890

[29] Balkwill F. Tumor necrosis factor and cancer. Nat Rev Cancer. (2009) 9:361–71. doi: 10.1038/nrc2628, PMID: 19343034

[30] Green DR. The end and after: how dying cells impact the living organism. Immunity. (2011) 35:441–4. doi: 10.1016/j.immuni.2011.10.003, PMID: 22035836

[31] Imamichi S Chen L Ito T Tong Y Onodera T Sasaki Y. Extracellular release of HMGB1 as an early potential biomarker for the therapeutic response in a xenograft model of boron neutron capture therapy. Biol (Basel). (2022) 11:420. doi: 10.3390/biology11030420, PMID: 35336794 PMC8945761

[32] Sato M Hirose K Takeno S Aihara T Nihei K Takai Y. Safety of boron neutron capture therapy with borofalan((10)B) and its efficacy on recurrent head and neck cancer: real-world outcomes from nationwide post-marketing surveillance. Cancers (Basel). (2024) 16:869. doi: 10.3390/cancers16050869, PMID: 38473231 PMC10931064

[33] Takeno S Yoshino Y Aihara T Higashino M Kanai Y Hu N. Preliminary outcomes of boron neutron capture therapy for head and neck cancers as a treatment covered by public health insurance system in Japan: Real-world experiences over a 2-year period. Cancer Med. (2024) 13:e7250. doi: 10.1002/cam4.7250, PMID: 38826090 PMC11145025

[34] Menéndez PR Roth BM Pereira MD Casal MR González SJ Feld DB. BNCT for skin melanoma in extremities: updated Argentine clinical results. Appl Radiat Isot. (2009) 67:S50–53. doi: 10.1016/j.apradiso.2009.03.020, PMID: 19375342

[35] Reva M Mendes M Sousa JJ Pais A Vitorino C. boron neutron capture therapy for glioblastoma: The delivery dilemma. Life Sci. (2025) 364:123435. doi: 10.1016/j.lfs.2025.123435, PMID: 39892861

[36] Papulino C Crepaldi M Favale G Del Gaudio N Benedetti R Nebbioso A. Aging and epigenetic implications in radiotherapy: The promise of BNCT. Ageing Res Rev. (2025) 110:102786. doi: 10.1016/j.arr.2025.102786, PMID: 40451443

[37] Shen S Wang S Zhou D Wu X Gao M Wu J. A clinician’s perspective on boron neutron capture therapy: promising advances, ongoing trials, and future outlook. Int J Radiat Biol. (2024) 100:1126–42. doi: 10.1080/09553002.2024.2373746, PMID: 38986056

[38] Cheng X Li F Liang L. Boron neutron capture therapy: clinical application and research progress. Curr Oncol. (2022) 29:7868–86. doi: 10.3390/curroncol29100622, PMID: 36290899 PMC9601095

[39] Kashihara T Mori T Nakaichi T Nakamura S Ito K Kurihara H. Correlation between L-amino acid transporter 1 expression and 4-borono-2-(18) F-fluoro-phenylalanine accumulation in humans. Cancer Med. (2023) 12:20564–72. doi: 10.1002/cam4.6635, PMID: 37881128 PMC10660410

[40] Lin KH Chen YW Wang LW Wang YF Hu LH Ting CH. Prognostic assessment of (18)F-boronophenylalanine positron emission tomography (BPA-PET) in salvage boron neutron capture therapy for Malignant brain tumors. Quant Imaging Med Surg. (2024) 14:4177–88. doi: 10.21037/qims-23-1769, PMID: 38846276 PMC11151257

[41] Chang CH Chen FH Wang LW Chiang CS. Circulating M-MDSC levels as an assessment marker for post-treatment tumor progression in recurrent HNC patients following radiation therapy: A case series. J Clin Med. (2024) 13:5130. doi: 10.3390/jcm13175130, PMID: 39274343 PMC11396399

[42] Chang CH Yu CF Chen FH Chen YW Chiang CS. The level of circulating M-MDSCs as an indicator for the therapeutic outcome of BNCT in end-stage Malignant brain tumor patients. Int J Part Ther. (2024) 14:100633. doi: 10.1016/j.ijpt.2024.100633, PMID: 39582734 PMC11585706

[43] Farr LE Sweet WH Robertson JS Foster CG Locksley HB Sutherland DL. Neutron capture therapy with boron in the treatment of glioblastoma multiforme. Am J Roentgenol Radium Ther Nucl Med. (1954) 71:279–93. 13124616

[44] Slatkin DN Stoner RD Rosander KM Kalef-Ezra JA Laissue JA. Central nervous system radiation syndrome in mice from preferential 10B(n, alpha)7Li irradiation of brain vasculature. Proc Natl Acad Sci U.S.A. (1988) 85:4020–4. doi: 10.1073/pnas.85.11.4020, PMID: 3375251 PMC280352

[45] Schwint AE Trivillin VA. Close-to-ideal’ tumor boron targeting for boron neutron capture therapy is possible with ‘less-than-ideal’ boron carriers approved for use in humans. Ther Delivery. (2015) 6:269–72. doi: 10.4155/tde.14.108, PMID: 25853302

[46] Elowitz EH Bergland RM Coderre JA Joel DD Chadha M Chanana AD. Biodistribution of p-boronophenylalanine in patients with glioblastoma multiforme for use in boron neutron capture therapy. Neurosurgery. (1998) 42:463–468; discussion 468-469. doi: 10.1097/00006123-199803000-00004, PMID: 9526978

[47] Coderre JA Chanana AD Joel DD Elowitz EH Micca PL Nawrocky MM. Biodistribution of boronophenylalanine in patients with glioblastoma multiforme: boron concentration correlates with tumor cellularity. Radiat Res. (1998) 149:163–70. doi: 10.2307/3579926 9457896

[48] Goodman JH Yang W Barth RF Gao Z Boesel CP Staubus AE. Boron neutron capture therapy of brain tumors: biodistribution, pharmacokinetics, and radiation dosimetry sodium borocaptate in patients with gliomas. Neurosurgery. (2000) 47:608–621; discussion 621-602. doi: 10.1097/00006123-200009000-00016, PMID: 10981748

[49] Oloo SO Smith KM Vicente M. Multi-functional boron-delivery agents for boron neutron capture therapy of cancers. Cancers (Basel). (2023) 15:3277. doi: 10.3390/cancers15133277, PMID: 37444386 PMC10340061

[50] Barth RF Mi P Yang W. Boron delivery agents for neutron capture therapy of cancer. Cancer Commun (Lond). (2018) 38:35. doi: 10.1186/s40880-018-0299-7, PMID: 29914561 PMC6006782

[51] Barth RF Gupta N Kawabata S. Evaluation of sodium borocaptate (BSH) and boronophenylalanine (BPA) as boron delivery agents for neutron capture therapy (NCT) of cancer: an update and a guide for the future clinical evaluation of new boron delivery agents for NCT. Cancer Commun (Lond). (2024) 44:893–909. doi: 10.1002/cac2.12582, PMID: 38973634 PMC11337926

[52] Xu H Liu Ji Li R Lin J Gui L Wang Y. Novel promising boron agents for boron neutron capture therapy: Current status and outlook on the future. Coordination Chem Rev. (2024) 511:215795. doi: 10.1016/j.ccr.2024.215795

[53] Ailuno G Balboni A Caviglioli G Lai F Barbieri F Dellacasagrande I. Boron vehiculating nanosystems for neutron capture therapy in cancer treatment. Cells. (2022) 11:4029. doi: 10.3390/cells11244029, PMID: 36552793 PMC9776957

[54] Ahmad F. Boron nanocomposites for boron neutron capture therapy and in biomedicine: evolvement and challenges. Biomater Res. (2025) 29:145. doi: 10.34133/bmr.0145, PMID: 40008112 PMC11850861

[55] Xu S Yu Y Zhang B Zhu K Cheng Y Zhang T. Boron carbide nanoparticles for boron neutron capture therapy. RSC Adv. (2025) 15:10717–30. doi: 10.1039/d5ra00734h, PMID: 40196817 PMC11973571

[56] Chen J Dai Q Yang Q Bao X Zhou Y Zhong H. Therapeutic nucleus-access BNCT drug combined CD47-targeting gene editing in glioblastoma. J Nanobiotechnol. (2022) 20:102. doi: 10.1186/s12951-022-01304-0, PMID: 35246144 PMC8895533

[57] Kiyomiya K Matsuo S Kurebe M. Mechanism of specific nuclear transport of adriamycin: the mode of nuclear translocation of adriamycin-proteasome complex. Cancer Res. (2001) 61:2467–71., PMID: 11289116

[58] Alamón C Dávila B García MF Nievas S Dagrosa MA Thorp S. A potential boron neutron capture therapy agent selectively suppresses high-grade glioma: *in vitro* and *in vivo* exploration. Mol Pharm. (2023) 20:2702–13. doi: 10.1021/acs.molpharmaceut.3c00152, PMID: 37013916

[59] Sauerwein WAG Sancey L Hey-Hawkins E Kellert M Panza L Imperio D. Theranostics in boron neutron capture therapy. Life (Basel). (2021) 11:330. doi: 10.3390/life11040330, PMID: 33920126 PMC8070338

[60] Chen J Xu M Li Z Kong Z Cai J Wang C. A bis-boron amino acid for positron emission tomography and boron neutron capture therapy. Angew Chem Int Ed Engl. (2025) 64:e202413249. doi: 10.1002/anie.202413249, PMID: 39349362

[61] Sforzi J Lanfranco A Stefania R Alberti D Bitonto V Parisotto S. A novel pH sensitive theranostic PLGA nanoparticle for boron neutron capture therapy in mesothelioma treatment. Sci Rep. (2023) 13:620. doi: 10.1038/s41598-023-27625-0, PMID: 36635364 PMC9837127

[62] Huang W Pan Y Zhong T He S Qi Y Huang Y. Near-infrared (10)B-BODIPY for precise guidance of tracer imaging and treatment in boron neutron capture therapy. Chem Commun (Camb). (2025) 61:9079–82. doi: 10.1039/d5cc01671a, PMID: 40401390

[63] Kondo N Hirano F Temma T. Evaluation of 3-borono-l-phenylalanine as a water-soluble boron neutron capture therapy agent. Pharmaceutics. (2022) 14:1106. doi: 10.3390/pharmaceutics14051106, PMID: 35631692 PMC9143228

[64] Wang S Zhang Z Miao L Zhang J Tang F Teng M. Construction of targeted (10)B delivery agents and their uptake in gastric and pancreatic cancer cells. Front Oncol. (2023) 13:1105472. doi: 10.3389/fonc.2023.1105472, PMID: 36845737 PMC9947830

[65] Nomoto T Inoue Y Yao Y Suzuki M Kanamori K Takemoto H. Poly(vinyl alcohol) boosting therapeutic potential of p-boronophenylalanine in neutron capture therapy by modulating metabolism. Sci Adv. (2020) 6:eaaz1722. doi: 10.1126/sciadv.aaz1722, PMID: 32010792 PMC6976296

[66] Nomoto T Yao Y Inoue Y Suzuki M Kanamori K Takemoto H. Fructose-functionalized polymers to enhance therapeutic potential of p-boronophenylalanine for neutron capture therapy. J Control Release. (2021) 332:184–93. doi: 10.1016/j.jconrel.2021.02.021, PMID: 33636247

[67] Zhang Z Wang X Dai Q Qin Y Sun X Suzuki M. Peptide-functionalized gold nanoparticles for boron neutron capture therapy with the potential to use in Glioblastoma treatment. Pharm Dev Technol. (2024) 29:862–73. doi: 10.1080/10837450.2024.2406044, PMID: 39286881

[68] Takeuchi K Hattori Y Kawabata S Futamura G Hiramatsu R Wanibuchi M. Synthesis and evaluation of dodecaboranethiol containing kojic acid (KA-BSH) as a novel agent for boron neutron capture therapy. Cells. (2020) 9:1551. doi: 10.3390/cells9061551, PMID: 32630612 PMC7349888

[69] Mi P Yanagie H Dewi N Yen HC Liu X Suzuki M. Block copolymer-boron cluster conjugate for effective boron neutron capture therapy of solid tumors. J Control Release. (2017) 254:1–9. doi: 10.1016/j.jconrel.2017.03.036, PMID: 28336377

[70] Masunaga S Kasaoka S Maruyama K Nigg D Sakurai Y Nagata K. The potential of transferrin-pendant-type polyethyleneglycol liposomes encapsulating decahydrodecaborate-(10)B (GB-10) as (10)B-carriers for boron neutron capture therapy. Int J Radiat Oncol Biol Phys. (2006) 66:1515–22. doi: 10.1016/j.ijrobp.2006.08.028, PMID: 17126210

[71] Barth RF Yang W Bartus RT Moeschberger ML Goodman JH. Enhanced delivery of boronophenylalanine for neutron capture therapy of brain tumors using the bradykinin analog Cereport (Receptor-Mediated Permeabilizer-7). Neurosurgery. (1999) 44:351–9. doi: 10.1097/00006123-199902000-00062, PMID: 9932889

[72] Barth RF Yang W Rotaru JH Moeschberger ML Boesel CP Soloway AH. Boron neutron capture therapy of brain tumors: enhanced survival and cure following blood-brain barrier disruption and intracarotid injection of sodium borocaptate and boronophenylalanine. Int J Radiat Oncol Biol Phys. (2000) 47:209–18. doi: 10.1016/s0360-3016(00)00421-1, PMID: 10758326

[73] Barth RF Yang W Rotaru JH Moeschberger ML Joel DD Nawrocky MM. Boron neutron capture therapy of brain tumors: enhanced survival following intracarotid injection of either sodium borocaptate or boronophenylalanine with or without blood-brain barrier disruption. Cancer Res. (1997) 57:1129–36. 9067283

[74] Yang W Barth RF Bartus RT Rotaru JH Moeschberger ML Ferketich AK. Improved survival after boron neutron capture therapy of brain tumors by Cereport-mediated blood-brain barrier modulation to enhance delivery of boronophenylalanine. Neurosurgery. (2000) 47:189–97. doi: 10.1097/00006123-200007000-00039, PMID: 10917362

[75] Yang W Barth RF Carpenter DE Moeschberger ML Goodman JH. Enhanced delivery of boronophenylalanine for neutron capture therapy by means of intracarotid injection and blood-brain barrier disruption. Neurosurgery. (1996) 38:985–92. doi: 10.1097/00006123-199605000-00027, PMID: 8727825

[76] Yang W Barth RF Rotaru JH Moeschberger ML Joel DD Nawrocky MM. Enhanced survival of glioma bearing rats following boron neutron capture therapy with blood-brain barrier disruption and intracarotid injection of boronophenylalanine. J Neurooncol. (1997) 33:59–70. doi: 10.1023/a:1005769214899, PMID: 9151224

[77] Yang W Barth RF Rotaru JH Moeschberger ML Joel DD Nawrocky MM. Boron neutron capture therapy of brain tumors: enhanced survival following intracarotid injection of sodium borocaptate with or without blood-brain barrier disruption. Int J Radiat Oncol Biol Phys. (1997) 37:663–72. doi: 10.1016/s0360-3016(96)00082-x, PMID: 9112465

[78] Liu Q Lv L Li H Liang H Hu F Su W. Microneedle-adapted PAMAM-BSH delivery facilitates spatiotemporal matching for melanoma boron neutron capture therapy. J Control Release. (2025) 384:113863. doi: 10.1016/j.jconrel.2025.113863, PMID: 40383156

[79] Kusaka S Voulgaris N Onishi K Ueda J Saito S Tamaki S. Therapeutic effect of boron neutron capture therapy on boronophenylalanine administration via cerebrospinal fluid circulation in glioma rat models. Cells. (2024) 13:1610. doi: 10.3390/cells13191610, PMID: 39404374 PMC11475075

[80] Li J Wang X Wang Z Zhao Y Zhang Z Li L. A transdermal drug delivery system based on dissolving microneedles for boron neutron capture therapy of melanoma. Biomater Sci. (2023) 11:7568–78. doi: 10.1039/d3bm01262j, PMID: 37861462

[81] Olaiz N Monti Hughes A Pozzi ECC Thorp S Curotto P Trivillin VA. Enhancement in the therapeutic efficacy of *in vivo* BNCT mediated by GB-10 with electroporation in a model of oral cancer. Cells. (2023) 12:1241. doi: 10.3390/cells12091241, PMID: 37174642 PMC10177359

[82] Kusaka S Morizane Y Tokumaru Y Tamaki S Maemunah IR Akiyama Y. Boron delivery to brain cells via cerebrospinal fluid (CSF) circulation for BNCT in a rat melanoma model. Biol (Basel). (2022) 11:397. doi: 10.3390/biology11030397, PMID: 35336771 PMC8945851

[83] Garabalino MA Olaiz N Portu A Saint Martin G Thorp SI Pozzi ECC. Electroporation optimizes the uptake of boron-10 by tumor for boron neutron capture therapy (BNCT) mediated by GB-10: a boron biodistribution study in the hamster cheek pouch oral cancer model. Radiat Environ Biophys. (2019) 58:455–67. doi: 10.1007/s00411-019-00796-z, PMID: 31123853

[84] Fan CH Wang TW Hsieh YK Wang CF Gao Z Kim A. Enhancing boron uptake in brain glioma by a boron-polymer/microbubble complex with focused ultrasound. ACS Appl Mater Interfaces. (2019) 11:11144–56. doi: 10.1021/acsami.8b22468, PMID: 30883079

[85] Wu CY Chan PC Chou LS Chang CW Yang FY Liu RS. Pulsed-focused ultrasound enhances boron drug accumulation in a human head and neck cancer xenograft-bearing mouse model. Mol Imaging Biol. (2014) 16:95–101. doi: 10.1007/s11307-013-0675-2, PMID: 23925592

[86] Khan AA Maitz C Quanyu C Hawthorne F. BNCT induced immunomodulatory effects contribute to mammary tumor inhibition. PloS One. (2019) 14:e0222022. doi: 10.1371/journal.pone.0222022, PMID: 31479484 PMC6719824

[87] Kawabata S Miyatake S Kajimoto Y Kuroda Y Kuroiwa T Imahori Y. The early successful treatment of glioblastoma patients with modified boron neutron capture therapy. Report of two cases. J Neurooncol. (2003) 65:159–65. doi: 10.1023/b:neon.0000003751.67562.8e, PMID: 14686736

[88] Barth RF Wu G Vicente M Grecula JC Gupta N. Boron neutron capture therapy of cancer: where do we stand now? Cancer Commun (Lond). (2024) 44:889–92. doi: 10.1002/cac2.12581, PMID: 38973667 PMC11337919

[89] Diaz AZ. Assessment of the results from the phase I/II boron neutron capture therapy trials at the Brookhaven National Laboratory from a clinician’s point of view. J Neurooncol. (2003) 62:101–9. doi: 10.1007/bf02699937, PMID: 12749706

[90] Chanana AD Capala J Chadha M Coderre JA Diaz AZ Elowitz EH. Boron neutron capture therapy for glioblastoma multiforme: interim results from the phase I/II dose-escalation studies. Neurosurgery. (1999) 44:1182–1192; discussion 1192-1183. doi: 10.1097/00006123-199906000-00013, PMID: 10371617

[91] Mishima Y Imahori Y Honda C Hiratsuka J Ueda S Ido T. *In vivo* diagnosis of human Malignant melanoma with positron emission tomography using specific melanoma-seeking 18F-DOPA analogue. J Neurooncol. (1997) 33:163–9. doi: 10.1023/a:1005746020350, PMID: 9151233

[92] Imahori Y Ueda S Ohmori Y Sakae K Kusuki T Kobayashi T. Positron emission tomography-based boron neutron capture therapy using boronophenylalanine for high-grade gliomas: part I. Clin Cancer Res. (1998) 4:1825–32. 9717808

[93] Kawabata S Miyatake S Nonoguchi N Hiramatsu R Iida K Miyata S. Survival benefit from boron neutron capture therapy for the newly diagnosed glioblastoma patients. Appl Radiat Isot. (2009) 67:S15–18. doi: 10.1016/j.apradiso.2009.03.015, PMID: 19398348

[94] Teng YC Chen J Zhong WB Liu YH. Correcting for the heterogeneous boron distribution in a tumor for BNCT dose calculation. Sci Rep. (2023) 13:15741. doi: 10.1038/s41598-023-42284-x, PMID: 37735579 PMC10514037

[95] Sato T Masunaga SI Kumada H Hamada N. Microdosimetric modeling of biological effectiveness for boron neutron capture therapy considering intra- and intercellular heterogeneity in (10)B distribution. Sci Rep. (2018) 8:988. doi: 10.1038/s41598-017-18871-0, PMID: 29343841 PMC5772701

[96] García-Montaño LA Licón-Muñoz Y Martinez FJ Keddari YR Ziemke MK Chohan MO. Dissecting intra-tumor heterogeneity in the glioblastoma microenvironment using fluorescence-guided multiple sampling. Mol Cancer Res. (2023) 21:755–67. doi: 10.1158/1541-7786.mcr-23-0048, PMID: 37255362 PMC10390891

[97] Bedard PL Hansen AR Ratain MJ Siu LL. Tumor heterogeneity in the clinic. Nature. (2013) 501:355–64. doi: 10.1038/nature12627, PMID: 24048068 PMC5224525

[98] Fisher R Pusztai L Swanton C. Cancer heterogeneity: implications for targeted therapeutics. Br J Cancer. (2013) 108:479–85. doi: 10.1038/bjc.2012.581, PMID: 23299535 PMC3593543

[99] da Costa JB Gibb EA Nykopp TK Mannas M Wyatt AW Black PC. Molecular tumor heterogeneity in muscle invasive bladder cancer: Biomarkers, subtypes, and implications for therapy. Urol Oncol. (2022) 40:287–94. doi: 10.1016/j.urolonc.2018.11.015, PMID: 30528886

[100] Fukuda H Honda C Wadabayashi N Kobayashi T Yoshino K Hiratsuka J. Pharmacokinetics of 10B-p-boronophenylalanine in tumors, skin and blood of melanoma patients: a study of boron neutron capture therapy for Malignant melanoma. Melanoma Res. (1999) 9:75–83. doi: 10.1097/00008390-199902000-00010, PMID: 10338337

[101] Liberman SJ Dagrosa A Jiménez Rebagliati RA Bonomi MR Roth BM Turjanski L. Biodistribution studies of boronophenylalanine-fructose in melanoma and brain tumor patients in Argentina. Appl Radiat Isot. (2004) 61:1095–100. doi: 10.1016/j.apradiso.2004.05.013, PMID: 15308198

[102] Tamura Y Miyatake S Nonoguchi N Miyata S Yokoyama K Doi A. Boron neutron capture therapy for recurrent Malignant meningioma. Case Rep J Neurosurg. (2006) 105:898–903. doi: 10.3171/jns.2006.105.6.898, PMID: 17405262

[103] Heber EM Trivillin VA Nigg DW Itoiz ME Gonzalez BN Rebagliati RJ. Homogeneous boron targeting of heterogeneous tumors for boron neutron capture therapy (BNCT): chemical analyses in the hamster cheek pouch oral cancer model. Arch Oral Biol. (2006) 51:922–9. doi: 10.1016/j.archoralbio.2006.03.015, PMID: 16696934

[104] Masunaga SI Sanada Y Tano K Sakurai Y Tanaka H Takata T. An attempt to improve the therapeutic effect of boron neutron capture therapy using commonly employed 10B-carriers based on analytical studies on the correlation among quiescent tumor cell characteristics, tumor heterogeneity and cancer stemness. J Radiat Res. (2020) 61:876–85. doi: 10.1093/jrr/rraa048, PMID: 32601693 PMC7674684

[105] Masunaga SI Ono K Kirihata M Takagaki M Sakurai Y Kinashi Y. Evaluation of the potential of p-boronophenylalaninol as a boron carrier in boron neutron capture therapy, referring to the effect on intratumor quiescent cells. Jpn J Cancer Res. (2001) 92:996–1007. doi: 10.1111/j.1349-7006.2001.tb01191.x, PMID: 11572769 PMC5926838

[106] Masunaga S Ono K Kirihata M Takagaki M Sakurai Y Kinashi Y. Potential of alpha-amino alcohol p-boronophenylalaninol as a boron carrier in boron neutron capture therapy, regarding its enantiomers. J Cancer Res Clin Oncol. (2003) 129:21–8. doi: 10.1007/s00432-002-0397-3, PMID: 12618897 PMC12161898

[107] Yoshida F Matsumura A Shibata Y Yamamoto T Nakauchi H Okumura M. Cell cycle dependence of boron uptake from two boron compounds used for clinical neutron capture therapy. Cancer Lett. (2002) 187:135–41. doi: 10.1016/s0304-3835(02)00380-4, PMID: 12359361

[108] Matsuya Y Sato T Kusumoto T Yachi Y Seino R Miwa M. Cell-cycle dependence on the biological effects of boron neutron capture therapy and its modification by polyvinyl alcohol. Sci Rep. (2024) 14:16696. doi: 10.1038/s41598-024-67041-6, PMID: 39030350 PMC11271528

[109] Yu LY Hsu CH Li CY Hong SY Chen CR Chen CS. Evaluating the biological effectiveness of boron neutron capture therapy by using microfluidics-based pancreatic tumor spheroids. Analyst. (2023) 148:3045–56. doi: 10.1039/d2an01812h, PMID: 37272284

[110] Jain RK. Normalizing tumor microenvironment to treat cancer: bench to bedside to biomarkers. J Clin Oncol. (2013) 31:2205–18. doi: 10.1200/jco.2012.46.3653, PMID: 23669226 PMC3731977

[111] Cesca M Morosi L Berndt A Fuso Nerini I Frapolli R Richter P. Bevacizumab-induced inhibition of angiogenesis promotes a more homogeneous intratumoral distribution of paclitaxel, improving the antitumor response. Mol Cancer Ther. (2016) 15:125–35. doi: 10.1158/1535-7163.mct-15-0063, PMID: 26494857

[112] Liu J Liao S Diop-Frimpong B Chen W Goel S Naxerova K. TGF-β blockade improves the distribution and efficacy of therapeutics in breast carcinoma by normalizing the tumor stroma. Proc Natl Acad Sci U.S.A. (2012) 109:16618–23. doi: 10.1073/pnas.1117610109, PMID: 22996328 PMC3478596

[113] Papageorgis P Polydorou C Mpekris F Voutouri C Agathokleous E Kapnissi-Christodoulou CP. Tranilast-induced stress alleviation in solid tumors improves the efficacy of chemo- and nanotherapeutics in a size-independent manner. Sci Rep. (2017) 7:46140. doi: 10.1038/srep46140, PMID: 28393881 PMC5385877

[114] Loeffler M Krüger JA Niethammer AG Reisfeld RA. Targeting tumor-associated fibroblasts improves cancer chemotherapy by increasing intratumoral drug uptake. J Clin Invest. (2006) 116:1955–62. doi: 10.1172/jci26532, PMID: 16794736 PMC1481657

[115] Provenzano PP Cuevas C Chang AE Goel VK Von Hoff DD Hingorani SR. Enzymatic targeting of the stroma ablates physical barriers to treatment of pancreatic ductal adenocarcinoma. Cancer Cell. (2012) 21:418–29. doi: 10.1016/j.ccr.2012.01.007, PMID: 22439937 PMC3371414

[116] Miyatake SI Wanibuchi M Hu N Ono K. Boron neutron capture therapy for Malignant brain tumors. J Neurooncol. (2020) 149:1–11. doi: 10.1007/s11060-020-03586-6, PMID: 32676954

[117] Kawabata S Miyatake S Kuroiwa T Yokoyama K Doi A Iida K. Boron neutron capture therapy for newly diagnosed glioblastoma. J Radiat Res. (2009) 50:51–60. doi: 10.1269/jrr.08043, PMID: 18957828

[118] Perona M Majdalani ME Rodríguez C Nievas S Carpano M Rossini A. Experimental studies of boron neutron capture therapy (BNCT) using histone deacetylase inhibitor (HDACI) sodium butyrate, as a complementary drug for the treatment of poorly differentiated thyroid cancer (PDTC). Appl Radiat Isot. (2020) 164:109297. doi: 10.1016/j.apradiso.2020.109297, PMID: 32768887

[119] Fukumura M Nonoguchi N Kawabata S Hiramatsu R Futamura G Takeuchi K. 5-Aminolevulinic acid increases boronophenylalanine uptake into glioma stem cells and may sensitize Malignant glioma to boron neutron capture therapy. Sci Rep. (2023) 13:10173. doi: 10.1038/s41598-023-37296-6, PMID: 37349515 PMC10287723

[120] Ohnishi K Misawa M Sikano N Nakai K Suzuki M. Enhancement of cancer cell-killing effects of boron neutron capture therapy by manipulating the expression of L-type amino acid transporter 1. Radiat Res. (2021) 196:17–22. doi: 10.1667/rade-20-00214.1, PMID: 33956158

[121] Dai L Liu J Yang T Yu X Lu Y Pan L. Lipoic acid-boronophenylalanine-derived multifunctional vesicles for cancer chemoradiotherapy. Nat Commun. (2025) 16:1329. doi: 10.1038/s41467-025-56507-4, PMID: 39900898 PMC11790874

[122] Wang LW Liu YH Chu PY Liu HM Peir JJ Lin KH. Boron neutron capture therapy followed by image-guided intensity-modulated radiotherapy for locally recurrent head and neck cancer: A prospective phase I/II trial. Cancers (Basel). (2023) 15:2762. doi: 10.3390/cancers15102762, PMID: 37345099 PMC10216174

[123] Fujimoto T Yamasaki O Kanehira N Matsushita H Sakurai Y Kenmotsu N. Overcoming immunotherapy resistance and inducing abscopal effects with boron neutron immunotherapy (B-NIT). Cancer Sci. (2024) 115:3231–47. doi: 10.1111/cas.16298, PMID: 39119813 PMC11447877

[124] Chiu YL Fu WY Huang WY Hsu FT Chen HW Wang TW. Enhancing cancer therapy: boron-rich polyboronate ester micelles for synergistic boron neutron capture therapy and PD-1/PD-L1 checkpoint blockade. Biomater Res. (2024) 28:40. doi: 10.34133/bmr.0040, PMID: 38933089 PMC11205919

[125] Li L Zhao Q Chen Z Zhao Z Du B Wang M. Size-tunable boron nanoreactors for boron neutron capture synergistic chemodynamic therapy of tumor. Adv Healthc Mater. (2025) 14:e2402307. doi: 10.1002/adhm.202402307, PMID: 39555631

[126] Li J Chen Z Wang Z Lv L Liu Q Chang Y. (10)Boron-doped carbon nanoparticles as delivery platforms for boron neutron capture therapy and photothermal therapy. Biomater Sci. (2025) 13:3280–97. doi: 10.1039/d5bm00068h, PMID: 40302467

[127] Fenech M Kirsch-Volders M Natarajan AT Surralles J Crott JW Parry J. Molecular mechanisms of micronucleus, nucleoplasmic bridge and nuclear bud formation in mammalian and human cells. Mutagenesis. (2011) 26:125–32. doi: 10.1093/mutage/geq052, PMID: 21164193

[128] Zierhut C Funabiki H. Regulation and consequences of cGAS activation by self-DNA. Trends Cell Biol. (2020) 30:594–605. doi: 10.1016/j.tcb.2020.05.006, PMID: 32546434 PMC7368801

[129] Krysko DV Garg AD Kaczmarek A Krysko O Agostinis P Vandenabeele P. Immunogenic cell death and DAMPs in cancer therapy. Nat Rev Cancer. (2012) 12:860–75. doi: 10.1038/nrc3380, PMID: 23151605

[130] Galluzzi L Vitale I Warren S Adjemian S Agostinis P Martinez AB. Consensus guidelines for the definition, detection and interpretation of immunogenic cell death. J Immunother Cancer. (2020) 8:e000337. doi: 10.1136/jitc-2019-000337, PMID: 32209603 PMC7064135

[131] Trivillin VA Langle YV Palmieri MA Pozzi ECC Thorp SI Benitez Frydryk DN. Evaluation of local, regional and abscopal effects of Boron Neutron Capture Therapy (BNCT) combined with immunotherapy in an ectopic colon cancer model. Br J Radiol. (2021) 94:20210593. doi: 10.1259/bjr.20210593, PMID: 34520668 PMC8631031

[132] Frydryk Benitez DN Palmieri MA Langle YV Monti Hughes A Pozzi ECC Thorp SI. Therapeutic efficacy, radiotoxicity and abscopal effect of BNCT at the RA-3 nuclear reactor employing oligo-fucoidan and glutamine as adjuvants in an ectopic colon cancer model in rats. Life (Basel). (2023) 13:1538. doi: 10.3390/life13071538, PMID: 37511913 PMC10381875

[133] Dovedi SJ Adlard AL Lipowska-Bhalla G McKenna C Jones S Cheadle EJ. Acquired resistance to fractionated radiotherapy can be overcome by concurrent PD-L1 blockade. Cancer Res. (2014) 74:5458–68. doi: 10.1158/0008-5472.can-14-1258, PMID: 25274032

[134] Deng S Hu L Chen G Ye J Xiao Z Guan T. A PD-L1 siRNA-loaded boron nanoparticle for targeted cancer radiotherapy and immunotherapy. Adv Mater. (2025) 37:e2419418. doi: 10.1002/adma.202419418, PMID: 39955653

[135] Tu X Qin B Zhang Y Zhang C Kahila M Nowsheen S. PD-L1 (B7-H1) competes with the RNA exosome to regulate the DNA damage response and can be targeted to sensitize to radiation or chemotherapy. Mol Cell. (2019) 74:1215–1226.e1214. doi: 10.1016/j.molcel.2019.04.005, PMID: 31053471 PMC6737939

[136] Zhang R Yang Y Dong W Lin M He J Zhang X. D-mannose facilitates immunotherapy and radiotherapy of triple-negative breast cancer via degradation of PD-L1. Proc Natl Acad Sci U.S.A. (2022) 119:e2114851119. doi: 10.1073/pnas.2114851119, PMID: 35181605 PMC8872783

[137] Shen W Pei P Zhang C Li J Han X Liu T. A polymeric hydrogel to eliminate programmed death-ligand 1 for enhanced tumor radio-immunotherapy. ACS Nano. (2023) 17:23998–4011. doi: 10.1021/acsnano.3c08875, PMID: 37988029

[138] Chang CH Chen CJ Yu CF Tsai HY Chen FH Chiang CS. Targeting M-MDSCs enhances the therapeutic effect of BNCT in the 4-NQO-induced murine head and neck squamous cell carcinoma model. Front Oncol. (2023) 13:1263873. doi: 10.3389/fonc.2023.1263873, PMID: 37886177 PMC10598372

[139] Feng M Jiang W Kim BYS Zhang CC Fu YX Weissman IL. Phagocytosis checkpoints as new targets for cancer immunotherapy. Nat Rev Cancer. (2019) 19:568–86. doi: 10.1038/s41568-019-0183-z, PMID: 31462760 PMC7002027

[140] Sharma B Kanwar SS. Phosphatidylserine: A cancer cell targeting biomarker. Semin Cancer Biol. (2018) 52:17–25. doi: 10.1016/j.semcancer.2017.08.012, PMID: 28870843

[141] Yang Y Zhao Z You C Pang M Zhong T Li Q. Lyophilized tumor cell-loaded (10)B-doped carbon dots for simultaneous boron neutron capture therapy and enhancement of antitumor immunity of prostate cancer. J Mater Chem B. (2025) 13:6701–11. doi: 10.1039/d5tb00478k, PMID: 40396385

[142] Chen K Liu S Lv L Tong J Chen J Liang H. Well-established immunotherapy with R837-loaded boron neutron capture-shocked tumor cells. Nano Today. (2023) 52:101995. doi: 10.1016/j.nantod.2023.101995

[143] Pashine A Valiante NM Ulmer JB. Targeting the innate immune response with improved vaccine adjuvants. Nat Med. (2005) 11:S63–68. doi: 10.1038/nm1210, PMID: 15812492

[144] Shi Y Guo Z Fu Q Shen X Zhang Z Sun W. Localized nuclear reaction breaks boron drug capsules loaded with immune adjuvants for cancer immunotherapy. Nat Commun. (2023) 14:1884. doi: 10.1038/s41467-023-37253-x, PMID: 37019890 PMC10076324

