# Supplementary material for: Boron neutron capture therapy in the context of tumor heterogeneity: progress, challenges, and future perspectives
Source: Front Oncol. 2025 Oct 17;15:1601013. doi: 10.3389/fonc.2025.1601013 (PMC12575145; doi:10.3389/fonc.2025.1601013)
Supplement: Supplementary file 1 [file Table1.docx]

Table 1 Clinical and preclinical applications of BNCT in tumors other than central nervous system, head and neck, and melanoma

| Year | Type of disease | Number of patients or tumor model | Boron delivery agents | Administration method | Therapeutic outcomes | Adverse effects | Refs. |
| --- | --- | --- | --- | --- | --- | --- | --- |
| 2007 | Hepatocellular carcinoma | 1 | BPA and BSH | Intra-arterial administration | One month after BNCT, the size of the liver tumors remained stable | Temporary temperature elevation to 38.3°C; the AST and ALT higher than 200 IU/l | (1) |
| 2014 | Recurrent hepatocellular carcinoma | 1 | BSH-WOW | Intra-arterial injection | 3 months: stable disease; 7 months: died of pulmonary metastasis-induced pneumonia | No adverse effects | (2) |
| 2012 | Locally recurrent lung cancer in the chest wall | 1 | BPA | Intravenous drip infusion | 7 months: significant tumor regression; 8 months: local recurrence at the margin of the BNCT field | No severe acute or late adverse events | (3) |
| 2008 | Diffuse or multiple pleural tumors | Malignant pleural mesothelioma 1; malignant short spindle cell tumor 1 | BPA | Intravenous drip infusion | The tumors regressed or remained stable in size for 3-6 months following BNCT | Radiation pneumonitis; radiation dermatitis | (4) |
| 2010 | Recurrent Malignant Peripheral Nerve Sheath Tumor | 1 | BPA | Intravenous injection | 1 year: 25% tumor size reduction; 18 months: no 18F-FDG uptake in residual mass; 2 years: no evidence of recurrence | Temporary dysphagia | (5) |
| 2015 | Malignant Peripheral Nerve Sheath Tumors | 2 patients; HS-Sch-2 xenograft-bearing nude mice | BPA | Intravenous injection | Tumor suppression in both clinical cases and animal models | Patiens: acute adverse event to skin, Grade 1. Animals: no damage to the surrounding normal tissues | (6) |
| 2024 | malignant peripheral nerve sheath tumor | 1 | BPA | Intravenous injection | Significant tumor shrinkage after BNCT, but tumor recurred 3.5 months later | Mild dysphagia and grade 2 anorexia | (7) |
| 2012 | Extramammary Paget's Disease | 2 | Not mentioned | Not mentioned | Complete tumor regression; 1 year: no recurrence or metastasis | Not mentioned | (8) |
| 2018 | Vulvar melanoma and genital extramammary Paget’s disease | Vulvar melanoma 1; genital extramammary Paget’s disease 3 | BPA | Intravenous drip infusion | Complete response within 6 months; no local recurrences during follow-up (1.1 to 6.9 years) | Moderate skin erosion, dysuria or contact pain | (9) |
| 2025 | Recurrent gastrointestinal cancers | Gastric cancer 1; rectal cancer 2; sigmoid colon cancer 1 | BPA | Intravenous injection | One patient achieved partial response and three patients demonstrated stable disease | Skin edema, oral erosion and throat pain or discomfort | (10) |
| 2014 | Radiation-induced osteosarcoma | 1 | BPA | Intravenous injection | 3 weeks: tumor significantly reduced; stable gait restored. 3 months: tumor further reduced; PET scan showed decreased metabolic activity. | Cerebral edema | (11) |
| 2014 | Osteosarcoma of the Temporomandibular Joint | 1 | Not mentioned | Not mentioned | 14 months: no signs of recurrence | Not mentioned | (12) |
| 2020 | Clear cell sarcoma of tendons and aponeuroses | 1 | BPA | Intravenous drip infusion | Total eradication of the tumor | Slight edema and plantar skin desquamation | (13) |
| 2021 | Synovial sarcoma | 1 | BPA | Intravenous drip infusion | Eight months after the two BNCT treatments, the tumor regressed, with a loss of FDG avidity | Grade 1 erythema of the skin at the irradiation site | (14) |
| 2022 | Scalp angiosarcoma | 2 | BPA | Intravenous drip infusion | 6 months: complete remission; one patient with extra-field tumor recurrence 20 months after BNCT | Hyperamylasemia, alopecia, nausea, et al | (15) |
| 2025 | cutaneous angiosarcoma and malignant melanoma | cutaneous angiosarcoma 9; malignant melanoma 1 | BPA | Intravenous drip infusion | 70% overall response rate within 180 days | Transient asymptomatic increase in serum amylase level, radiation dermatitis and alopecia | (16) |
| 2009 | Diffuse liver metastases | 2 | BPA | Intravenous drip infusion | Complete necrosis of the tumor; one died at 33 days post-treatment due to cardiomyopathy; the other died at 44 months post-treatment due to recurrence | Post-irradiation syndrome | (17) |
| 2025 | Left axillary lymph node metastasis of breast cancer | 1 | BPA | Intravenous injection | Tumor shrinkage and pain relief | No significant adverse reactions | (18) |
| 2007 | Malignant tumors spreading in the thoracic cavity, mimicking malignant pleural mesothelioma | SCCVII syngeneic C3H/He mice | BPA | Intraperitoneal injection | a significantly longer survival time in BNCT group than in the control group (31 days vs. 7 days) | Minimal lung fibrosis | (19) |
| 2014 | Clear Cell Sarcoma | HS-MM xenograft-bearing nude mice, MP-CCS-SY xenograft-bearing nude mice and SU-CCS-1 xenograft-bearing nude mice | BPA | Intravenous injection | The tumor volume significantly decreased until 20 days after neutron irradiation, after which it gradually increased. | No damage to the surrounding normal tissues | (20) |
| 2023 | Osteosarcoma | UMR-106 syngeneic SD rats | BPA | Intravenous injection | Effectively controlled the growth of the orthotopic osteosarcoma | Weight loss in the short term after treatment | (21) |
| 2025 | Myxofibrosarcoma | MFS xenograft-bearing nude mice | BPA | Intravenous injection | Inhibition of tumor growth | None observed | (22) |
| 2025 | Epithelioid Sarcoma | VA-ES-BJ xenograft-bearing nude mice | BPA | Intravenous injection | Tumor volume significantly decreased in BNCT group | No skin damage or other adverse reactions | (23) |
| 2022 | Hepatocellular Carcinoma | HepG2 and HepG2-R | BA | treated with BA for 30 minutes in the cell culture medium | More effective than γ-ray in killing HepG2-R cells | Not mentioned | (24) |
| 2025 | Hepatocellular Carcinoma | Hepa1-6; HepG2; Hepa1-6 xenograft-bearing nude mice | BPA | Intravenous injection | Cell viability significantly decreased after BPA-BNCT irradiation, and the tumor growth inhibition rate in mice reached 77% | No tissue damage in vivo | (25) |
| 2022 | Colorectal cancer | SW-620 xenograft-bearing SCID mice | BPA or BSH | Intravenous injection | The tumor growth in the BNCT group slowed down significantly | No significant adverse reactions | (26) |
| 2022 | Colorectal cancer | DLD-1 xenograft-bearing nude mice | BPA | Intraperitoneal injection | Prolonged survival in BNCT group mice | No significant adverse effects | (27) |
| 2023 | Cervical cancer | SiHa and Hela | BPA | treated with BPA in the cell culture medium | BNCT effectively killed SiHa and HeLa cells | Not mentioned | (28) |
| 2025 | Gastrointestinal stromal tumors | GIST-T1 and GIST-T1/IM-R; GIST-T1 xenograft-bearing SCID mice | BPA | Treated with BPA in the cell culture medium; Intraperitoneal injection | BPA-mediated BNCT exhibited significant antitumor activity both in vitro and in vivo | Temporary weight loss of the animal models | (29) |
| 2019 | Lung metastases of colon carcinoma | DHD/K12/TRb syngeneic BDIX rats | BPA or BPA+ GB-10 | Intravenous injection | Suppression of tumor growth; extension of survival time | Alopecia, edema, erythema, and moist desquamation | (30) |
| 2020 | Bone Metastasis from Breast Cancer | MDA-MB-231-luc xenograft-bearing nude mice | BPA | Intravenous injection | Inhibition of tumor growth in cases of intramedullary small tumors; no antitumor effects in cases with pathological fractures | Not mentioned | (31) |

1. Suzuki M, Sakurai Y, Hagiwara S, Masunaga S, Kinashi Y, Nagata K, et al. First attempt of boron neutron capture therapy (BNCT) for hepatocellular carcinoma. *Jpn J Clin Oncol* (2007) 37: 376-381. <https://doi.org/10.1093/jjco/hym039>

2. Yanagie H, Higashi S, Seguchi K, Ikushima I, Fujihara M, Nonaka Y, et al. Pilot clinical study of boron neutron capture therapy for recurrent hepatic cancer involving the intra-arterial injection of a (10)BSH-containing WOW emulsion. *Appl Radiat Isot* (2014) 88: 32-37. <https://doi.org/10.1016/j.apradiso.2014.01.014>

3. Suzuki M, Suzuki O, Sakurai Y, Tanaka H, Kondo N, Kinashi Y, et al. Reirradiation for locally recurrent lung cancer in the chest wall with boron neutron capture therapy (BNCT). *International Cancer Conference Journal* (2012) 1: 235-238. <https://doi.org/10.1007/s13691-012-0048-8>

4. Suzuki M, Endo K, Satoh H, Sakurai Y, Kumada H, Kimura H, et al. A novel concept of treatment of diffuse or multiple pleural tumors by boron neutron capture therapy (BNCT). *Radiother Oncol* (2008) 88: 192-195. <https://doi.org/10.1016/j.radonc.2008.06.009>

5. Inoue M, Lee CM, Ono K, Suzuki M, Tokunaga T, Sawa Y, et al. Clinical effectiveness of boron neutron capture therapy for a recurrent malignant peripheral nerve sheath tumor in the mediastinum. *J Thorac Oncol* (2010) 5: 2037-2038. <https://doi.org/10.1097/JTO.0b013e3181f1cd86>

6. Fujimoto T, Andoh T, Sudo T, Fujita I, Fukase N, Takeuchi T, et al. Potential of boron neutron capture therapy (BNCT) for malignant peripheral nerve sheath tumors (MPNST). *Appl Radiat Isot* (2015) 106: 220-225. <https://doi.org/10.1016/j.apradiso.2015.07.059>

7. Watanabe Y, Chen YW, Igaki H, Arakawa A, Tao K, Sugiyama M, et al. Boron neutron capture therapy prolongs survival in a patient with a recurrent malignant peripheral nerve sheath tumor-A case report. *Pediatr Blood Cancer* (2024) 71: e31011. <https://doi.org/10.1002/pbc.31011>

8. E. Makino, S. Sasaoka, T. Aihara, Y. Sakurai, A. Maruhashi, K. Ono4, et al. The First Clinical Trial of Boron Neutron Capture Therapy Using 10B-para-boronophenylalanine for Treating Extramammary Paget’s Disease. *european journal of cancer* (2012) 48: S244-S245.

9. Hiratsuka J, Kamitani N, Tanaka R, Yoden E, Tokiya R, Suzuki M, et al. Boron neutron capture therapy for vulvar melanoma and genital extramammary Paget's disease with curative responses. *Cancer Commun (Lond)* (2018) 38: 38. <https://doi.org/10.1186/s40880-018-0297-9>

10. Yanagie H, Maruyama S, Oyama K, Ono Y, Kuroyama S, Nonaka Y, et al. First Experiences of Pilot Clinical Studies on Boron Neutron Capture Therapy for Recurrent Gastrointestinal Cancers Using an Intravenous Injection of (10)BPA. *In Vivo* (2025) 39: 1470-1491. <https://doi.org/10.21873/invivo.13948>

11. Futamura G, Kawabata S, Siba H, Kuroiwa T, Suzuki M, Kondo N, et al. A case of radiation-induced osteosarcoma treated effectively by boron neutron capture therapy. *Radiat Oncol* (2014) 9: 237. <https://doi.org/10.1186/s13014-014-0237-z>

12. Uchiyama Y, Matsumoto K, Murakami S, Kanesaki T, Matsumoto A, Kishino M, et al. MRI in a case of osteosarcoma in the temporomandibular joint. *Dentomaxillofac Radiol* (2014) 43: 20130280. <https://doi.org/10.1259/dmfr.20130280>

13. Fujimoto T, Suzuki M, Sudo T, Fujita I, Sakuma T, Sakurai Y, et al. Boron neutron capture therapy for clear cell sarcoma. *Appl Radiat Isot* (2020) 166: 109324. <https://doi.org/10.1016/j.apradiso.2020.109324>

14. Fujimoto T, Suzuki M, Kuratsu S, Fujita I, Morishita M, Sudo T, et al. BNCT for primary synovial sarcoma. *Appl Radiat Isot* (2021) 169: 109407. <https://doi.org/10.1016/j.apradiso.2020.109407>

15. Igaki H, Murakami N, Nakamura S, Yamazaki N, Kashihara T, Takahashi A, et al. Scalp angiosarcoma treated with linear accelerator-based boron neutron capture therapy: A report of two patients. *Clin Transl Radiat Oncol* (2022) 33: 128-133. <https://doi.org/10.1016/j.ctro.2022.02.006>

16. Kashihara T, Nakamura S, Yamazaki N, Takahashi A, Namikawa K, Ogata D, et al. Boron neutron capture therapy for cutaneous angiosarcoma and malignant melanoma: First in-human phase I clinical trial. *Radiother Oncol* (2025) 202: 110607. <https://doi.org/10.1016/j.radonc.2024.110607>

17. Zonta A, Pinelli T, Prati U, Roveda L, Ferrari C, Clerici AM, et al. Extra-corporeal liver BNCT for the treatment of diffuse metastases: what was learned and what is still to be learned. *Appl Radiat Isot* (2009) 67: S67-75. <https://doi.org/10.1016/j.apradiso.2009.03.087>

18. Fujimoto T, Maekawa Y, Hori S, Oguro A, Hori A, Fujita I, et al. Boron neutron capture therapy (BNCT) for left axillary lymph node metastasis of recurrent breast cancer. *Appl Radiat Isot* (2025) 219: 111715. <https://doi.org/10.1016/j.apradiso.2025.111715>

19. Suzuki M, Sakurai Y, Masunaga S, Kinashi Y, Nagata K, Maruhashi A, et al. A preliminary experimental study of boron neutron capture therapy for malignant tumors spreading in thoracic cavity. *Jpn J Clin Oncol* (2007) 37: 245-249. <https://doi.org/10.1093/jjco/hym022>

20. Andoh T, Fujimoto T, Sudo T, Suzuki M, Sakurai Y, Sakuma T, et al. Boron neutron capture therapy as new treatment for clear cell sarcoma: trial on different animal model. *Appl Radiat Isot* (2014) 88: 59-63. <https://doi.org/10.1016/j.apradiso.2013.12.007>

21. Hsu CF, Liu HM, Peir JJ, Liao JW, Chen KS, Chen YW, et al. Therapeutic Efficacy and Radiobiological Effects of Boric-Acid-Mediated BNCT in an Osteosarcoma-Bearing SD Rat Model. *Life (Basel)* (2023) 13: <https://doi.org/10.3390/life13020514>

22. Fujimoto T, Andoh T, Sudo T, Fujita I, Sakurai Y, Takata T, et al. Potential of boron neutron capture therapy (BNCT) for myxofibrosarcoma. *Appl Radiat Isot* (2025) 217: 111603. <https://doi.org/10.1016/j.apradiso.2024.111603>

23. Fujimoto T, Andoh T, Sudo T, Sakuma T, Fujita I, Sakurai Y, et al. Potential of boron neutron capture therapy (BNCT) for epithelioid sarcoma. *Appl Radiat Isot* (2025) 222: 111846. <https://doi.org/10.1016/j.apradiso.2025.111846>

24. Huang CY, Lai ZY, Hsu TJ, Chou FI, Liu HM,Chuang YJ. Boron Neutron Capture Therapy Eliminates Radioresistant Liver Cancer Cells by Targeting DNA Damage and Repair Responses. *J Hepatocell Carcinoma* (2022) 9: 1385-1401. <https://doi.org/10.2147/jhc.s383959>

25. Zhang T, Zhang P, Zhang H, Zhang Z, Jin X, Zhao T, et al. Pharmacokinetic, biodistribution, safety and efficacy studies of borophenylalanine (BPA) in BNCT in hepatocellular carcinoma cells and tumor-bearing mouse model. *Sci Rep* (2025) 15: 29212. <https://doi.org/10.1038/s41598-025-14885-1>

26. Kanygin VV, Kasatova AI, Zavjalov EL, Razumov IA, Kolesnikov SI, Kichigin AI, et al. Effects of Boron Neutron Capture Therapy on the Growth of Subcutaneous Xenografts of Human Colorectal Adenocarcinoma SW-620 in Immunodeficient Mice. *Bull Exp Biol Med* (2022) 172: 359-363. <https://doi.org/10.1007/s10517-022-05392-8>

27. Arima J, Taniguchi K, Yamamoto M, Watanabe T, Suzuki Y, Hamamoto H, et al. Anti-tumor effect of boron neutron capture therapy in pelvic human colorectal cancer in a mouse model. *Biomed Pharmacother* (2022) 154: 113632. <https://doi.org/10.1016/j.biopha.2022.113632>

28. Terada S, Tsunetoh S, Tanaka Y, Tanaka T, Kashiwagi H, Takata T, et al. Boron uptake of boronophenylalanine and the effect of boron neutron capture therapy in cervical cancer cells. *Appl Radiat Isot* (2023) 197: 110792. <https://doi.org/10.1016/j.apradiso.2023.110792>

29. Hagihara S, Arima J, Ueda Y, Inomata Y, Shima T, Suzuki M, et al. Evaluating boron neutron capture therapy as a potential treatment for unresectable gastrointestinal stromal tumors. *Gastric Cancer* (2025) 28: 924-934. <https://doi.org/10.1007/s10120-025-01633-7>

30. Trivillin VA, Serrano A, Garabalino MA, Colombo LL, Pozzi EC, Hughes AM, et al. Translational boron neutron capture therapy (BNCT) studies for the treatment of tumors in lung. *Int J Radiat Biol* (2019) 95: 646-654. <https://doi.org/10.1080/09553002.2019.1564080>

31. Andoh T, Fujimoto T, Satani R, Suzuki M, Wada K, Sudo T, et al. Preclinical study of boron neutron capture therapy for bone metastasis using human breast cancer cell lines. *Appl Radiat Isot* (2020) 165: 109257. <https://doi.org/10.1016/j.apradiso.2020.109257>
