# Supplementary material for: Boron neutron capture therapy in the context of tumor heterogeneity: progress, challenges, and future perspectives
Source: Front Oncol. 2025 Oct 17;15:1601013. doi: 10.3389/fonc.2025.1601013 (PMC12575145; doi:10.3389/fonc.2025.1601013)
Supplement: Supplementary file 2 [file Table2.docx]

Table 2 Summary of active tumor-targeting boron delivery agents (BDAs) in the last decade

| Cellular localization | Ligand | Target | Name of BDAs and references |
| --- | --- | --- | --- |
| Cell membrane | Folate | Folate Receptor | B4C-APTES-PEG2K-FA (1); PBC-IP (2, 3); 10B-Fe3O4Nzyme-CS-FA-PEG (4); Carborane-Containing Folic Acid bis-Amides (5); PBCs (6); ND-BSH complex (7); PBC (8); FA-3PCB (9); PLGA-NP-Folate (10) |
|  | RGD peptide | αvβ3 integrin | c(RGD-BPA-K)(PEG2-(4-iodophenylbutyl)) (11); AuNPs-BSH&PEG-cRGD (12); cRGD(D-BPA)K (13); cRGD-MID-AC (14); cRGD-COS-CB/PTX (15); closo-dodecaborate-(Ga-DOTA)-c(RGDfK) and iodo-closo-dodecaborate-(Ga-DOTA)-c(RGDfK) (16); DOX-CB@lipo-pDNA-iRGD (17); cRGD-MID-BSA (18); iRGD-PEG-PCCL-B (19); 10BSGRF NPs (20) |
|  | Anti-EGFR antibody; EGFR-TKI; EGFR-targeted ASO; nucleotide; lapatinib | EGFR | B4C anti-EGFR (21); hybrid 1 (22); anti-EGFR-Gd^10^B_6_ NPs (23); anti-EGFR-10BPO4 NPs (24); B-ASOs (25)；Z33-DB (26); NBEs (27); lap-BPA (28); Closo-Carboranyl- and Metallacarboranyl [1,2,3]triazolyl-Decorated Lapatinib-Scaffold (29) |
|  | Amino acid | LAT1 | BTS (30); PEG-P[Lys/Lys(fructose)]-BPA (31); B139 (32); TB-5-BT, DBA-5-BT, TB-6-BT and AZ-2,7-DBT (33); FBY (34); ACBC-BSH (35) |
|  | Glucose | GLU1 | compound 4 (36); B-Glc (37); Glucose-BSH (38)； 6-O-(o-carboranylmethyl)-D-glucopyranoside (39) |
|  | HA | CD44 | BS-CyP-NPs-HA (40); hyaluronic acid-based nanogels (41); HA/CBP-H (42); Carborane-TAT@HA nanomicelles (43); HA-BND-S (44) |
|  | Anti-HER-2 antibody; lapatinib | HER-2 | BNNT/β-glucan-IgG complex (45); HER-2-iLMI-CB (46); 61-B-AuNPs (47); lap-BPA (28); Closo-Carboranyl- and Metallacarboranyl [1,2,3]triazolyl-Decorated Lapatinib-Scaffold (29) |
|  | Anti-LDLR antibody; LDL | LDLR | B4C anti-LDLR (21); AT101/LDL (48) |
|  | Apo | SCARA5 | Apo:[o-COSAN]^−^ (49) |
|  | Angiopep-2 | LPR1 | ANG-B (50) |
|  | Transferrin | Transferrin receptor | 10BSH-TF-PEG-DSBL (51) |
|  | IF7 peptide | Anxa1 | IF7C(10BPA)RR and IF7K(10BSH)RR (52) |
|  | Galactose residue | ASGP-R | MPEG-b-P(LA-co-D-Gal-MPCB) (53) |
|  | Biotin | Biotin receptor | BBC-IP (54) |
|  | oligopeptide | PEPT1 | BPA-Tyr and Tyr-BPA (55) |
|  | sBB2L | GRPR | [L-Gal_2_-Cb]_8_-Dap^3^-EG_3_-sBB2L (56) |
|  | GHRP-6 and Ipamorelin | GhrR | HwAWfK(5)-NH2 and Aib-H-(D-2-Nal)-fK(5)-NH2 (57) |
| Cytoplasm / Organelles | DPA; indole carboxamides | TSPO | DPA-BSTPG (58, 59); carboranyl – indole carboxamides (60) |
|  | SP94 peptide | GRP78 | SP94-LB@BA-MSN (61); |
|  | Ureidosulfamido group; sulfamido group | CAIX | ^10^B-NO2-CA-USF and ^0^B-F-CA-USF (62); CA-SF and ^0^B-CA-SF (63) |
|  | Fenbufen | COX-2 | FBPin (64) |
| Cell nucleus | PBA | Cell nucleus | PBA (65) |
|  | DOX | Cell nucleus | DOX-CB@lipo-pDNA-iRGD (17) |
|  | Deoxycytidine | Cell nucleus | N(4)-[B-(4,4,5,5-tetramethyl-1,3,2-dioxaborolan)methyl]-2′-deoxycytidine (66) |
| Extracellular / Secreted | FPBA; PBA; BPA | SA | FPBA-PRX (67); PBA-BDP (68); L^P^  B-3 vesicles (69); PBA (65); Nano^PBA^ (70); BPA@CSSU-PNI (71) |
|  | HSA | SPARC | BCDs-HSA (72) |
|  | MMP inhibitor | MMP | 1,4-triazole 1 (B1), 1,5-triazole 2 (B2), CGS-27023A analog 3 and CGS-27023A analog 1,4-triazole 4 (73) |
|  | Sunitinib | VEGFR | Hybrid 1 (74) |
| Tumor microenvironment | Nitroimidazole group | Hypoxic tumor regions | B139 (32); B-381 (75) |
|  | pHLIP | Acidic tumor microenvironment | SZGO:Cr-10B-NF-pHLIP (76) |
|  | FAPI | FAP | Carborane-FAPI (77) |

1. Xu S, Yu Y, Zhang B, Zhu K, Cheng Y,Zhang T. Boron carbide nanoparticles for boron neutron capture therapy. *RSC Adv* (2025) 15: 10717-10730. <https://doi.org/10.1039/d5ra00734h>

2. Miura K, Nishimura K, Suzuki M,Nakamura H. Boron neutron capture therapy with pteroyl-closo-dodecaborate-conjugated 4-(p-iodophenyl)butyric acid (PBC-IP) for a head and neck squamous cell carcinoma (SAS) model mice. *Appl Radiat Isot* (2025) 221: 111837. <https://doi.org/10.1016/j.apradiso.2025.111837>

3. Nishimura K, Kashiwagi H, Morita T, Fukuo Y, Okada S, Miura K, et al. Efficient neutron capture therapy of glioblastoma with pteroyl-closo-dodecaborate-conjugated 4-(p-iodophenyl)butyric acid (PBC-IP). *J Control Release* (2023) 360: 249-259. <https://doi.org/10.1016/j.jconrel.2023.06.022>

4. Zhang T, Xu D, Chen M, Wang H, Wang Y, Wang C, et al. Boron-Containing Nanoenzyme for Effective Treatment of Melanoma through Boron Neutron Capture Therapy Combined with Catalytic Therapy. *ACS Appl Mater Interfaces* (2025) 17: 46720-46732. <https://doi.org/10.1021/acsami.5c10501>

5. Gruzdev DA, Telegina AA, Levit GL, Solovieva OI, Gusel'nikova TY, Razumov IA, et al. Carborane-Containing Folic Acid bis-Amides: Synthesis and In Vitro Evaluation of Novel Promising Agents for Boron Delivery to Tumour Cells. *Int J Mol Sci* (2022) 23: <https://doi.org/10.3390/ijms232213726>

6. Nakagawa F, Kawashima H, Morita T,Nakamura H. Water-Soluble closo-Docecaborate-Containing Pteroyl Derivatives Targeting Folate Receptor-Positive Tumors for Boron Neutron Capture Therapy. *Cells* (2020) 9: <https://doi.org/10.3390/cells9071615>

7. Matsumoto Y, Hattori K, Arima H, Motoyama K, Higashi T, Ishikawa H, et al. Folate-appended cyclodextrin improves the intratumoral accumulation of existing boron compounds. *Appl Radiat Isot* (2020) 163: 109201. <https://doi.org/10.1016/j.apradiso.2020.109201>

8. Kanemitsu T, Kawabata S, Fukumura M, Futamura G, Hiramatsu R, Nonoguchi N, et al. Folate receptor-targeted novel boron compound for boron neutron capture therapy on F98 glioma-bearing rats. *Radiat Environ Biophys* (2019) 58: 59-67. <https://doi.org/10.1007/s00411-018-0765-2>

9. Singh A, Kim BK, Mackeyev Y, Rohani P, Mahajan SD, Swihart MT, et al. Boron-Nanoparticle-Loaded Folic-Acid-Functionalized Liposomes to Achieve Optimum Boron Concentration for Boron Neutron Capture Therapy of Cancer. *J Biomed Nanotechnol* (2019) 15: 1714-1723. <https://doi.org/10.1166/jbn.2019.2800>

10. Alberti D, Protti N, Franck M, Stefania R, Bortolussi S, Altieri S, et al. Theranostic Nanoparticles Loaded with Imaging Probes and Rubrocurcumin for Combined Cancer Therapy by Folate Receptor Targeting. *ChemMedChem* (2017) 12: 502-509. <https://doi.org/10.1002/cmdc.201700039>

11. Bibi I, Kang KJ, Kim JY, Mushtaq S,Park JA. Development of Structurally Identical Therapeutic and Diagnostic Agents for Image-Guided Boron Neutron Capture Therapy: c(RGD-BPA-K) Peptide with (125)I/(nat)I Albumin-Binding Moiety. *Mol Pharm* (2025) 22: 3423-3432. <https://doi.org/10.1021/acs.molpharmaceut.5c00291>

12. Zhang Z, Wang X, Dai Q, Qin Y, Sun X, Suzuki M, et al. Peptide-functionalized gold nanoparticles for boron neutron capture therapy with the potential to use in Glioblastoma treatment. *Pharm Dev Technol* (2024) 29: 862-873. <https://doi.org/10.1080/10837450.2024.2406044>

13. Kim S, Mushtaq S, Lee KC, Park JA,Kim JY. (64)Cu-Labeled Boron-Containing Cyclic RGD Peptides for BNCT and PET Imaging. *ACS Med Chem Lett* (2024) 15: 344-348. <https://doi.org/10.1021/acsmedchemlett.4c00007>

14. Tsujino K, Kashiwagi H, Nishimura K, Kayama R, Yoshimura K, Fukuo Y, et al. Improved Boron Neutron Capture Therapy Using Integrin αvβ3-Targeted Long-Retention-Type Boron Carrier in a F98 Rat Glioma Model. *Biology (Basel)* (2023) 12: <https://doi.org/10.3390/biology12030377>

15. Yang Q, Dai Q, Bao X, Zhou Y, Lu Y, Zhong H, et al. Evaluation of a Tumor-Targeting Oligosaccharide Nanosystem in BNCT on an Orthotopic Hepatocellular Carcinoma Model. *Mol Pharm* (2023) 20: 1025-1038. <https://doi.org/10.1021/acs.molpharmaceut.2c00771>

16. Mishiro K, Imai S, Ematsu Y, Hirose K, Fuchigami T, Munekane M, et al. RGD Peptide-Conjugated Dodecaborate with the Ga-DOTA Complex: A Preliminary Study for the Development of Theranostic Agents for Boron Neutron Capture Therapy and Its Companion Diagnostics. *J Med Chem* (2022) 65: 16741-16753. <https://doi.org/10.1021/acs.jmedchem.2c01586>

17. Chen J, Dai Q, Yang Q, Bao X, Zhou Y, Zhong H, et al. Therapeutic nucleus-access BNCT drug combined CD47-targeting gene editing in glioblastoma. *J Nanobiotechnology* (2022) 20: 102. <https://doi.org/10.1186/s12951-022-01304-0>

18. Kawai K, Nishimura K, Okada S, Sato S, Suzuki M, Takata T, et al. Cyclic RGD-Functionalized closo-Dodecaborate Albumin Conjugates as Integrin Targeting Boron Carriers for Neutron Capture Therapy. *Mol Pharm* (2020) 17: 3740-3747. <https://doi.org/10.1021/acs.molpharmaceut.0c00478>

19. Chen J, Yang Q, Liu M, Lin M, Wang T, Zhang Z, et al. Remarkable Boron Delivery Of iRGD-Modified Polymeric Nanoparticles For Boron Neutron Capture Therapy. *Int J Nanomedicine* (2019) 14: 8161-8177. <https://doi.org/10.2147/ijn.s214224>

20. Kuthala N, Vankayala R, Li YN, Chiang CS,Hwang KC. Engineering Novel Targeted Boron-10-Enriched Theranostic Nanomedicine to Combat against Murine Brain Tumors via MR Imaging-Guided Boron Neutron Capture Therapy. *Adv Mater* (2017) 29: <https://doi.org/10.1002/adma.201700850>

21. Rudawska A, Szermer-Olearnik B, Szczygieł A, Mierzejewska J, Węgierek-Ciura K, Żeliszewska P, et al. Functionalized Boron Carbide Nanoparticles as Active Boron Delivery Agents Dedicated to Boron Neutron Capture Therapy. *Int J Nanomedicine* (2025) 20: 6637-6657. <https://doi.org/10.2147/ijn.s516534>

22. Alamón C, Dávila B, García MF, Nievas S, Dagrosa MA, Thorp S, et al. A Potential Boron Neutron Capture Therapy Agent Selectively Suppresses High-Grade Glioma: In Vitro and in Vivo Exploration. *Mol Pharm* (2023) 20: 2702-2713. <https://doi.org/10.1021/acs.molpharmaceut.3c00152>

23. Shanmugam M, Kuthala N, Kong X, Chiang CS,Hwang KC. Combined Gadolinium and Boron Neutron Capture Therapies for Eradication of Head-and-Neck Tumor Using Gd(10)B(6) Nanoparticles under MRI/CT Image Guidance. *JACS Au* (2023) 3: 2192-2205. <https://doi.org/10.1021/jacsau.3c00250>

24. Kuthala N, Shanmugam M, Yao CL, Chiang CS,Hwang KC. One step synthesis of (10)B-enriched (10)BPO(4) nanoparticles for effective boron neutron capture therapeutic treatment of recurrent head-and-neck tumor. *Biomaterials* (2022) 290: 121861. <https://doi.org/10.1016/j.biomaterials.2022.121861>

25. Kaniowski D, Suwara J, Ebenryter-Olbińska K, Jakóbik-Kolon A,Nawrot B. EGFR-Targeted Cellular Delivery of Therapeutic Nucleic Acids Mediated by Boron Clusters. *Int J Mol Sci* (2022) 23: <https://doi.org/10.3390/ijms232314793>

26. Nakase I, Aoki A, Sakai Y, Hirase S, Ishimura M, Takatani-Nakase T, et al. Antibody-Based Receptor Targeting Using an Fc-Binding Peptide-Dodecaborate Conjugate and Macropinocytosis Induction for Boron Neutron Capture Therapy. *ACS Omega* (2020) 5: 22731-22738. <https://doi.org/10.1021/acsomega.0c01377>

27. Wang M, Tong Y, Luo Q,Hu S. Comparative Study on Neutron Irradiation Sensitization Effects of Nucleotide Borate Esters and Several Other Boron Agents. *Radiat Res* (2020) 193: 249-262. <https://doi.org/10.1667/rr15473.1>

28. Wang S, Zhang Z, Miao L, Zhang J, Tang F, Teng M, et al. Construction of targeted (10)B delivery agents and their uptake in gastric and pancreatic cancer cells. *Front Oncol* (2023) 13: 1105472. <https://doi.org/10.3389/fonc.2023.1105472>

29. Couto M, Alamón C, García MF, Kovacs M, Trias E, Nievas S, et al. Closo-Carboranyl- and Metallacarboranyl [1,2,3]triazolyl-Decorated Lapatinib-Scaffold for Cancer Therapy Combining Tyrosine Kinase Inhibition and Boron Neutron Capture Therapy. *Cells* (2020) 9: <https://doi.org/10.3390/cells9061408>

30. Raitano A, Martin T, Zhang C, Malinao MC, Capo L, Ikeura M, et al. Boronotyrosine, a Borylated Amino Acid Mimetic with Enhanced Solubility, Tumor Boron Delivery, and Retention for the Re-emerging Boron Neutron Capture Therapy Field. *J Med Chem* (2023) 66: 13809-13820. <https://doi.org/10.1021/acs.jmedchem.3c01265>

31. Nomoto T, Yao Y, Inoue Y, Suzuki M, Kanamori K, Takemoto H, et al. Fructose-functionalized polymers to enhance therapeutic potential of p-boronophenylalanine for neutron capture therapy. *J Control Release* (2021) 332: 184-193. <https://doi.org/10.1016/j.jconrel.2021.02.021>

32. Li R, Zhang J, Guo J, Xu Y, Duan K, Zheng J, et al. Application of Nitroimidazole-Carbobane-Modified Phenylalanine Derivatives as Dual-Target Boron Carriers in Boron Neutron Capture Therapy. *Mol Pharm* (2020) 17: 202-211. <https://doi.org/10.1021/acs.molpharmaceut.9b00898>

33. Chio CM, Huang YC, Chou YC, Hsu FC, Lai YB,Yu CS. Boron Accumulation in Brain Tumor Cells through Boc-Protected Tryptophan as a Carrier for Boron Neutron Capture Therapy. *ACS Med Chem Lett* (2020) 11: 589-596. <https://doi.org/10.1021/acsmedchemlett.0c00064>

34. Li J, Shi Y, Zhang Z, Liu H, Lang L, Liu T, et al. A Metabolically Stable Boron-Derived Tyrosine Serves as a Theranostic Agent for Positron Emission Tomography Guided Boron Neutron Capture Therapy. *Bioconjug Chem* (2019) 30: 2870-2878. <https://doi.org/10.1021/acs.bioconjchem.9b00578>

35. Futamura G, Kawabata S, Nonoguchi N, Hiramatsu R, Toho T, Tanaka H, et al. Evaluation of a novel sodium borocaptate-containing unnatural amino acid as a boron delivery agent for neutron capture therapy of the F98 rat glioma. *Radiat Oncol* (2017) 12: 26. <https://doi.org/10.1186/s13014-017-0765-4>

36. Imperio D, Postuma I, Villani S, Del Grosso E, Cansolino L, Ferrari C, et al. A Novel Closo-Ortho-Carborane-Based Glucosamine Derivative as a Promising Agent for Boron Neutron Capture Therapy. *Pharmaceuticals (Basel)* (2025) 18: <https://doi.org/10.3390/ph18070986>

37. Imlimthan S, Bahrami K, Pehkonen H, Centanni A, Montaser AB, Värä A, et al. Biological evaluation of a glucose-based boron carrier as a potential agent for boron neutron capture therapy. *Int J Cancer* (2025) <https://doi.org/10.1002/ijc.70054>

38. Fujimoto T, Teraishi F, Kanehira N, Tajima T, Sakurai Y, Kondo N, et al. BNCT pancreatic cancer treatment strategy with glucose-conjugated boron drug. *Biomaterials* (2024) 309: 122605. <https://doi.org/10.1016/j.biomaterials.2024.122605>

39. Matović J, Järvinen J, Bland HC, Sokka IK, Imlimthan S, Ferrando RM, et al. Addressing the Biochemical Foundations of a Glucose-Based "Trojan Horse"-Strategy to Boron Neutron Capture Therapy: From Chemical Synthesis to In Vitro Assessment. *Mol Pharm* (2020) 17: 3885-3899. <https://doi.org/10.1021/acs.molpharmaceut.0c00630>

40. Ye M, Li B, Shi W, Liu H, Wang Y, Chen W, et al. Preparation and tumor-targeting evaluation of BS-CyP albumin nanoparticles modified with hyaluronic acid based on boron neutron capture therapy. *J Biomed Mater Res A* (2023) 111: 1176-1184. <https://doi.org/10.1002/jbm.a.37506>

41. Coninx S, Kalot G, Godard A, Bodio E, Goze C, Sancey L, et al. Tailored hyaluronic acid-based nanogels as theranostic boron delivery systems for boron neutron cancer therapy. *Int J Pharm X* (2022) 4: 100134. <https://doi.org/10.1016/j.ijpx.2022.100134>

42. Yamana K, Kawasaki R, Sanada Y, Tabata A, Bando K, Yoshikawa K, et al. Tumor-targeting hyaluronic acid/fluorescent carborane complex for boron neutron capture therapy. *Biochem Biophys Res Commun* (2021) 559: 210-216. <https://doi.org/10.1016/j.bbrc.2021.04.037>

43. Quan H, Fan L, Huang Y, Xia X, He Y, Liu S, et al. Hyaluronic acid-decorated carborane-TAT conjugation nanomicelles: A potential boron agent with enhanced selectivity of tumor cellular uptake. *Colloids Surf B Biointerfaces* (2021) 204: 111826. <https://doi.org/10.1016/j.colsurfb.2021.111826>

44. Sasai M, Nakamura H, Sougawa N, Sakurai Y, Suzuki M,Lee CM. Novel Hyaluronan Formulation Enhances the Efficacy of Boron Neutron Capture Therapy for Murine Mesothelioma. *Anticancer Res* (2016) 36: 907-911.

45. Kawasaki R, Oshige A, Yamana K, Hirano H, Nishimura K, Miura Y, et al. HER-2-Targeted Boron Neutron Capture Therapy with Carborane-integrated Immunoliposomes Prepared via an Exchanging Reaction. *Chemistry* (2023) 29: e202302486. <https://doi.org/10.1002/chem.202302486>

46. Yamana K, Kawasaki R, Kondo K, Hirano H, Kawamura S, Sanada Y, et al. HER-2-targeted boron neutron capture therapy using an antibody-conjugated boron nitride nanotube/β-1,3-glucan complex. *Nanoscale Adv* (2023) 5: 3857-3861. <https://doi.org/10.1039/d3na00028a>

47. Wu CY, Lin JJ, Chang WY, Hsieh CY, Wu CC, Chen HS, et al. Development of theranostic active-targeting boron-containing gold nanoparticles for boron neutron capture therapy (BNCT). *Colloids Surf B Biointerfaces* (2019) 183: 110387. <https://doi.org/10.1016/j.colsurfb.2019.110387>

48. Alberti D, Deagostino A, Toppino A, Protti N, Bortolussi S, Altieri S, et al. An innovative therapeutic approach for malignant mesothelioma treatment based on the use of Gd/boron multimodal probes for MRI guided BNCT. *J Control Release* (2018) 280: 31-38. <https://doi.org/10.1016/j.jconrel.2018.04.043>

49. Alberti D, Piña Marcos JN, Rakhshan S, Protti N, Altieri S, Nuez-Martínez M, et al. Cobaltabis(dicarbollide) [o-COSAN](-) loaded apoferritin: an innovative high-capacity boron delivery system to target tumour cells for BNCT applications. *Nanoscale* (2025) 17: 11624-11633. <https://doi.org/10.1039/d5nr00362h>

50. Xiang J, Ma L, Tong J, Zuo N, Hu W, Luo Y, et al. Boron-peptide conjugates with angiopep-2 for boron neutron capture therapy. *Front Med (Lausanne)* (2023) 10: 1199881. <https://doi.org/10.3389/fmed.2023.1199881>

51. Yanagie H, Yanagawa M, Morishita Y, Shinohara A, Dewi N, Nonaka Y, et al. Suppression of Tumor Growth in a Rabbit Hepatic Cancer Model by Boron Neutron Capture Therapy With Liposomal Boron Delivery Systems. *In Vivo* (2021) 35: 3125-3135. <https://doi.org/10.21873/invivo.12607>

52. Yoneyama T, Hatakeyama S, Sutoh Yoneyama M, Yoshiya T, Uemura T, Ishizu T, et al. Tumor vasculature-targeted (10)B delivery by an Annexin A1-binding peptide boosts effects of boron neutron capture therapy. *BMC Cancer* (2021) 21: 72. <https://doi.org/10.1186/s12885-020-07760-x>

53. Zhang T, Li G, Li S, Wang Z, He D, Wang Y, et al. Asialoglycoprotein receptor targeted micelles containing carborane clusters for effective boron neutron capture therapy of hepatocellular carcinoma. *Colloids Surf B Biointerfaces* (2019) 182: 110397. <https://doi.org/10.1016/j.colsurfb.2019.110397>

54. Nishimura K, Tanaka S, Miura K, Okada S, Suzuki M,Nakamura H. A Water-Soluble Small Molecule Boron Carrier Targeting Biotin Receptors for Neutron Capture Therapy. *ACS Omega* (2024) 9: 51631-51640. <https://doi.org/10.1021/acsomega.4c09388>

55. Miyabe J, Ohgaki R, Saito K, Wei L, Quan L, Jin C, et al. Boron delivery for boron neutron capture therapy targeting a cancer-upregulated oligopeptide transporter. *J Pharmacol Sci* (2019) 139: 215-222. <https://doi.org/10.1016/j.jphs.2019.01.012>

56. Hoppenz P, Els-Heindl S, Kellert M, Kuhnert R, Saretz S, Lerchen HG, et al. A Selective Carborane-Functionalized Gastrin-Releasing Peptide Receptor Agonist as Boron Delivery Agent for Boron Neutron Capture Therapy. *J Org Chem* (2020) 85: 1446-1457. <https://doi.org/10.1021/acs.joc.9b02406>

57. Worm DJ, Els-Heindl S, Kellert M, Kuhnert R, Saretz S, Koebberling J, et al. A stable meta-carborane enables the generation of boron-rich peptide agonists targeting the ghrelin receptor. *J Pept Sci* (2018) 24: e3119. <https://doi.org/10.1002/psc.3119>

58. Kashiwagi H, Hattori Y, Kawabata S, Kayama R, Yoshimura K, Fukuo Y, et al. Multi-Targeted Neutron Capture Therapy Combined with an 18 kDa Translocator Protein-Targeted Boron Compound Is an Effective Strategy in a Rat Brain Tumor Model. *Cancers (Basel)* (2023) 15: <https://doi.org/10.3390/cancers15041034>

59. Hattori Y, Ishimura M, Ohta Y, Takenaka H, Kawabata S,Kirihata M. Dodecaborate Conjugates Targeting Tumor Cell Overexpressing Translocator Protein for Boron Neutron Capture Therapy. *ACS Med Chem Lett* (2022) 13: 50-54. <https://doi.org/10.1021/acsmedchemlett.1c00377>

60. Narlawar R, Austin CJD, Kahlert J, Selleri S, Da Pozzo E, Martini C, et al. Remarkable Enhancement in Boron Uptake Within Glioblastoma Cells With Carboranyl-Indole Carboxamides. *Chem Asian J* (2018) 13: 3321-3327. <https://doi.org/10.1002/asia.201801175>

61. Tang H, Wang Z, Hao H, Luo W, Yang J, Li M, et al. Boron-Containing Mesoporous Silica Nanoparticles with Effective Delivery and Targeting of Liver Cancer Cells for Boron Neutron Capture Therapy. *ACS Appl Mater Interfaces* (2024) 16: 22934-22945. <https://doi.org/10.1021/acsami.4c02897>

62. Lanfranco A, Rakhshan S, Alberti D, Renzi P, Zarechian A, Protti N, et al. Combining BNCT with carbonic anhydrase inhibition for mesothelioma treatment: Synthesis, in vitro, in vivo studies of ureidosulfamido carboranes. *Eur J Med Chem* (2024) 270: 116334. <https://doi.org/10.1016/j.ejmech.2024.116334>

63. Alberti D, Michelotti A, Lanfranco A, Protti N, Altieri S, Deagostino A, et al. In vitro and in vivo BNCT investigations using a carborane containing sulfonamide targeting CAIX epitopes on malignant pleural mesothelioma and breast cancer cells. *Sci Rep* (2020) 10: 19274. <https://doi.org/10.1038/s41598-020-76370-1>

64. Yeh CN, Chang CW, Chung YH, Tien SW, Chen YR, Chen TW, et al. Synthesis and characterization of boron fenbufen and its F-18 labeled homolog for boron neutron capture therapy of COX-2 overexpressed cholangiocarcinoma. *Eur J Pharm Sci* (2017) 107: 217-229. <https://doi.org/10.1016/j.ejps.2017.07.019>

65. Chen J, Li Y, Gan X, Weng C, Fang X,Liu G. Unlocking the potential: phenylboronic acid as a nuclear-targeting boron agent for neutron capture therapy. *Med Oncol* (2024) 41: 104. <https://doi.org/10.1007/s12032-024-02351-3>

66. Uram Ł, Nizioł J, Maj P, Sobich J, Rode W,Ruman T. N(4)-[B-(4,4,5,5-tetramethyl-1,3,2-dioxaborolan)methyl]-2'-deoxycytidine as a potential boron delivery agent with respect to glioblastoma. *Biomed Pharmacother* (2017) 95: 749-755. <https://doi.org/10.1016/j.biopha.2017.08.134>

67. Matsumoto Y, Arase H, Ishiki H, Takeuchi H, Sugawara Y, Taharabaru T, et al. Design and evaluation of a supramolecular boron compound using a cyclodextrin-based polyrotaxane for boron neutron capture therapy. *Carbohydr Polym* (2025) 354: 123343. <https://doi.org/10.1016/j.carbpol.2025.123343>

68. Huang W, Pan Y, Zhong T, He S, Qi Y,Huang Y. Near-infrared (10)B-BODIPY for precise guidance of tracer imaging and treatment in boron neutron capture therapy. *Chem Commun (Camb)* (2025) 61: 9079-9082. <https://doi.org/10.1039/d5cc01671a>

69. Dai L, Liu J, Yang T, Yu X, Lu Y, Pan L, et al. Lipoic acid-boronophenylalanine-derived multifunctional vesicles for cancer chemoradiotherapy. *Nat Commun* (2025) 16: 1329. <https://doi.org/10.1038/s41467-025-56507-4>

70. Kim A, Suzuki M, Matsumoto Y, Fukumitsu N,Nagasaki Y. Non-isotope enriched phenylboronic acid-decorated dual-functional nano-assembles for an actively targeting BNCT drug. *Biomaterials* (2021) 268: 120551. <https://doi.org/10.1016/j.biomaterials.2020.120551>

71. Soleimanbeigi M, Dousti F, Hassanzadeh F, Mirian M, Varshosaz J, Kasesaz Y, et al. Boron phenyl alanine targeted chitosan-PNIPAAm core-shell thermo-responsive nanoparticles: boosting drug delivery to glioblastoma in BNCT. *Drug Dev Ind Pharm* (2021) 47: 1607-1623. <https://doi.org/10.1080/03639045.2022.2032132>

72. Zhong T, Yang Y, Pang M, Pan Y, Jing S, Qi Y, et al. Human Serum Albumin-Coated (10)B Enriched Carbon Dots as Targeted "Pilot Light" for Boron Neutron Capture Therapy. *Adv Sci (Weinh)* (2024) 11: e2406577. <https://doi.org/10.1002/advs.202406577>

73. Flieger S, Takagaki M, Kondo N, Lutz MR, Jr., Gupta Y, Ueda H, et al. Carborane-Containing Hydroxamate MMP Ligands for the Treatment of Tumors Using Boron Neutron Capture Therapy (BNCT): Efficacy without Tumor Cell Entry. *Int J Mol Sci* (2023) 24: <https://doi.org/10.3390/ijms24086973>

74. Couto M, Alamón C, Nievas S, Perona M, Dagrosa MA, Teixidor F, et al. Bimodal Therapeutic Agents Against Glioblastoma, One of the Most Lethal Forms of Cancer. *Chemistry* (2020) 26: 14335-14340. <https://doi.org/10.1002/chem.202002963>

75. Luderer MJ, Muz B, de la Puente P, Chavalmane S, Kapoor V, Marcelo R, et al. A Hypoxia-Targeted Boron Neutron Capture Therapy Agent for the Treatment of Glioma. *Pharm Res* (2016) 33: 2530-2539. <https://doi.org/10.1007/s11095-016-1977-2>

76. Sharma KS, Raju MS, Phapale S, Valvi SK, Dubey AK, Goswami D, et al. Multimodal Applications of Zinc Gallate-Based Persistent Luminescent Nanoparticles in Cancer Treatment: Tumor Margining, Diagnosis, and Boron Neutron Capture Therapy. *ACS Appl Bio Mater* (2022) 5: 3134-3145. <https://doi.org/10.1021/acsabm.2c00081>

77. Zhang J, Wu Y, Lu W, Xiao Y, Liu S,Yu J. Carborane-FAPI conjugate: A potential FAP-targeted boron agent with improved boron content. *Appl Radiat Isot* (2024) 209: 111330. <https://doi.org/10.1016/j.apradiso.2024.111330>
