# Supplementary material for: Boron neutron capture therapy in the context of tumor heterogeneity: progress, challenges, and future perspectives
Source: Front Oncol. 2025 Oct 17;15:1601013. doi: 10.3389/fonc.2025.1601013 (PMC12575145; doi:10.3389/fonc.2025.1601013)
Supplement: Supplementary file 3 [file Table3.docx]

Table 3 Summary of theranostic boron delivery agents (BDAs) in the last decade

| Year | Name of BDA | Boron  source | Target | Imaging contrast agents | Imaging  technique | Cells and animal models | Characteristics | Refs. |
| --- | --- | --- | --- | --- | --- | --- | --- | --- |
| 2025 | BBPA | BA |  | 18F | PET | B16-F10 cells;  B16-F10, U-118MG, UM-SCC-22B, T-98G, GH3 and BGC-823 tumor models;  C57 mice | High tumor uptake; low toxicity;  superior to BPA at same dose | (1) |
| 2019 | FBY | trifluoroborate |  | 18F | PET | B16-F10 cells;  B16-F10 tumor models | High metabolic stability; tumor targeting; favorable biocompatibility; significant tumor growth inhibition and prolonged survival time in mice | (2) |
| 2022 | ¹⁹F-BPA | BPA |  | 19F | MRI | PANC-1 cells | Efficient uptake by pancreatic cancer cells; non-invasive assessment of boron biodistribution | (3) |
| 2025 | the B (^10^B)-Compound-Loaded AB-Lac Particles | Carb or diC6-Carb |  | ICG | Fluorescence imaging | AsPC-1 cells;  AsPC-1 tumor models | Accumulated in tumors to reach therapeutic 10B levels for BNCT; effectively suppressed tumor cell growth following neutron irradiation | (4) |
| 2025 | c(RGD-BPA-K)(PEG2-(4-iodophenylbutyl));  c(RGD-BPA-K)(PEG2-(4-[125I]iodophenylbutyl)) | BPA | αvβ3 integrin | 125I | SPECT/CT | U-87MG cells;  HEK-293 cells;  U-87MG tumor models | High tumor uptake; long retention;  elevated T/M ratio | (5) |
| 2019 | ^123^I-B-AuNPs, 1^23^I-61-B-AuNPs, and ^123^I-trastuzumab-B-AuNPs | BC-EG-SH | HER2 | 123I | microSPECT/CT | N87 cells;  N87 tumor models | High tumor targeting efficiency; high T/M ratio; monitorable via non-invasive imaging | (6) |
| 2025 | PBA-BDP | PBA | sialic acid receptors | BODIPY | Fluorescence imaging | RM-1 cells and B16-F10 cells;  L929 cells;  RM-1 and B16-F10 tumor models | Near-infrared fluorescence properties; tumor targeting；low toxicity；good biocompatibility；effectively inhibited tumor growth；significantly improved survival rate in tumor-bearing mice | (7) |
| 2022 | aza-BODIPY-BSH-loaded HA Nanogel | BSH | CD44 | aza-BODIPY | Fluorescence imaging | U-87MG cells;  HDMEC cells;  U-87MG tumor models | Good biocompatibility; tumor targeting；high 10B content; prolonged accumulation at the tumor site | (8) |
| 2020 | aza-SWIR-BSH-01 | BSH |  | aza-BODIPY | Fluorescence Imaging | U-251 MG and U-87 MG cells;  U-87 MG tumor models;  CAM models | Good water solubility; high tumor uptake; long retention time in tumor tissue; high T/M ratio; effectively inhibited the growth of U-251 MG tumors | (9) |
| 2024 | BCDs-HSA | BA | SPARC | BCDs | Fluorescence Imaging | RM-1 cells and B16-F10 cells;  L929 cells;  RM-1 and B16-F10 tumor models | High 10B content; good biocompatibility；low toxicity；excitation-independent fluorescent emission; tumor targetin；effectively inhibited tumor growth | (10) |
| 2024 | cRGD(d-BPA)K;  [64Cu]Cu-DOTA-cRGD(d-BPA)K | BPA | αvβ3 integrin | 64Cu | PET | U-87MG cells;  U-87MG tumor models | Good water solubility; tumor targeting; high T/B and T/M ratio; long retention | (11) |
| 2022 | Boronsome and inhibitor-loaded boronsome | Carborane |  | 64Cu | PET | 4T1 cells;  4T1 tumor models | High tumor accumulation; long tumor retention; low uptake in surrounding normal tissues; significant suppression of tumor growth; capacity for chemotherapy drug encapsulation | (12) |
| 2021 | AuNR-mPEG@[4]^−^ | COSAN |  | 64Cu | PET | MKN-45 cells;  HDFa;  MKN-45 tumor models | Good biocompatibility; accumulation in the tumor; effective killing of tumor cells after neutron irradiation; potential for PTT | (13) |
| 2020 | DSPE-BCOP-5T and ^64^Cu-DSPE-BCOP-5T | Carborane |  | 64Cu | PET | 4T1 Cells;  4T1 tumor models | Good biocompatibility and plasma stability; accumulation in tumor tissue; significant tumor suppression and prolonged survival in mice after neutron irradiation | (14) |
| 2018 | BPN and ^64^Cu-BPN | TBPP |  | TBPP;  ^64^Cu | Fluorescence Imaging;  PET | B16-F10 and 4T1cells;  B16-F10 and 4T1 tumor models | High tumor accumulation; high T/N ratio; long tumor retention; low toxicity; significant tumor suppression and prolonged survival time | (15) |
| 2024 | A-PInd-C | Carborane |  | PInd | Fluorescence Imaging | HeLa and HCT-116 cells;  L02 cells | Excellent pH-responsive drug release; superior cellular targeting and imaging capability; significant anti-tumor efficacy with good biocompatibility | (16) |
| 2023 | BS-CyP-NPs-HA | BSH | CD44 | CyP | Fluorescence imaging | 4T1 cells;  A549 cells;  LO2 cells;  4T1 tumor models | Excellent tumor targeting; good biocompatibility and safety; imaging potential | (17) |
| 2023 | HSA-Cy5-HcyTFAc-GCB12H11 and HSA-Cy7-HcyTFAc-GCB12H11 | Dodecaborane |  | Cy5 or Cy7; 19F | Fluorescence Imaging;  MRI | T98G cells | Enabled visualization within a living organism; prolonged the drug’s half-life in the bloodstream; provided both chemotherapy and BNCT effects | (18) |
| 2021 | HSA-Cy5-HcyTFAc-B12H11 and HSA-Cy5-HcyAc-B12H11-TTFA | B12H11 |  | Cy5;  19F | Fluorescence Imaging;  MRI | T98G and U-87 MG cells | Bimodal imaging capabilities; extended systemic circulation; significant reduction in survival following neutron irradiation | (19) |
| 2020 | cRGD-MID-BSA and Cy5-cRGD-MID-BSA | Dodecaborate | αvβ3 integrin | Cy5 | Fluorescence Imaging | U-87 MG and A549 cells;  Colon 26 cells;  U-87 MG tumor models;  Colon 26 tumor models | Low cytotoxicity; tumor targeting; selective tumor accumulation; significant antitumor activity | (20) |
| 2023 | Gd-MID-BSA | Dodecaborate |  | Gd | MRI | CT26 cells;  CT26 tumor models | MRI-guided non-invasive monitoring of boron distribution; enhanced tumor suppression via combined BNCT and GdNCT | (21) |
| 2023 | AT101/PLGA-His | Carborane |  | Gd | MRI | AB22 cells;  MeT-5A cells;  AB22 tumor models | pH-Sensitive; suitable for MRI-guided therapy; tumor targeting; effectively killed tumor cells | (22) |
| 2023 | Anti-EGFR-Gd10B6 NPs | BA | EGFR | Gd | MRI and CT | HTB-43 cells;  HUVECs;  HTB-43 tumor models | Effective dual-modal MRI/CT imaging guidance; low cytotoxicity; combined GdNCT effect for enhanced tumor cell killing, including cancer stem cells | (23) |
| 2020 | B-MSNs | Na₂[¹⁰B₁₂H₁₁SH] |  | Gd | MRI | CH-2879 cells;  ALDH+ cancer stem cells;  CH-2879 tumor models | Good biocompatibility; tumor accumulation; significant cytotoxicity against both chondrosarcoma cells and radioresistant cancer stem cells | (24) |
| 2018 | AT101/LDL | Carborane | LDLR | Gd | MRI | ZL34 cells;  AE17 cells;  MRC−5;  NMuMg;  ZL34 tumor models;  AE17 tumor models | Tumor targeting; high T/M ratio; significant tumor growth inhibition following neutron irradiation; potential of MRI-guided therapy | (25) |
| 2017 | ^10^BSGRF NPs | ^10^B-enriched  boron powder | αvβ3 integrin | FITC;  Gd | Fluorescence Imaging;  MRI | ALTS1C1 cells;  ALTS1C1 tumor models | Tumor targeting; high T/B ratio; significant tumor growth inhibition via neutron irradiation; dual-modal imaging | (26) |
| 2017 | PLGA-NP-Folate | RbCur | folate receptors | Gd | MRI | IGROV-1 and MCF-7 cells;  NMuMg and BALB/C 3T3 | Tumor targeting; improved therapeutic outcomes through combination of BNCT with curcumin; potential of MRI-guided therapy | (27) |
| 2022 | closo-dodecaborate-(Ga-DOTA)-c(RGDfK) (16) and 125I (17) | Dodecaborate | αvβ3 integrin | 67Ga | PET | U-87 MG cells;  U-87 MG tumor models;  ddY mice | High stability; tumor targeting; high tumor accumulation; low non-target uptake | (28) |
| 2022 | SZGO:Cr-10B-NF | BA | Acidic tumor environment | ZnGa_2_O_4_:Cr | photon imager | WEHI-164 and B16-F10 cells;  WEHI-164 and B16-F10 tumor models; | Persistent luminescence enabled clear tumor boundary identification; specific accumulation at the acidic tumor site via pHLIP conjugation; significant inhibition of tumor growth after neutron irradiation | (29) |
| 2021 | Fe-B NPs | Elemental boron |  | Fe | MRI | 4T1 and B16 cells;  L929 cells;  PBMCs,  Healthy Balb/c Mice | Magnetic properties for magnetic accumulation and hyperthermia; high boron content; good biocompatibility; applicability of MRI-guided BNCT | (30) |
| 2021 | HA/CBP-H complex | carborane | CD44 | CBP-H | Fluorescence imaging | Colon26 cells;  RAW 264.7 | High boron concentration; tumor targeting; fluorescent properties; BNCT efficacy equal to or greater than that of the clinical agent BPA-fructose | (31) |

1. Chen J, Xu M, Li Z, Kong Z, Cai J, Wang C, et al. A Bis-Boron Amino Acid for Positron Emission Tomography and Boron Neutron Capture Therapy. *Angew Chem Int Ed Engl* (2025) 64: e202413249. <https://doi.org/10.1002/anie.202413249>

2. Li J, Shi Y, Zhang Z, Liu H, Lang L, Liu T, et al. A Metabolically Stable Boron-Derived Tyrosine Serves as a Theranostic Agent for Positron Emission Tomography Guided Boron Neutron Capture Therapy. *Bioconjug Chem* (2019) 30: 2870-2878. <https://doi.org/10.1021/acs.bioconjchem.9b00578>

3. Ciardiello A, Altieri S, Ballarini F, Bocci V, Bortolussi S, Cansolino L, et al. Multimodal evaluation of (19)F-BPA internalization in pancreatic cancer cells for boron capture and proton therapy potential applications. *Phys Med* (2022) 94: 75-84. <https://doi.org/10.1016/j.ejmp.2021.12.011>

4. Fithroni AB, Inoue H, Zhou S, Hakim TFN, Tada T, Suzuki M, et al. Novel Drug Delivery Particles Can Provide Dual Effects on Cancer "Theranostics" in Boron Neutron Capture Therapy. *Cells* (2025) 14: <https://doi.org/10.3390/cells14010060>

5. Bibi I, Kang KJ, Kim JY, Mushtaq S,Park JA. Development of Structurally Identical Therapeutic and Diagnostic Agents for Image-Guided Boron Neutron Capture Therapy: c(RGD-BPA-K) Peptide with (125)I/(nat)I Albumin-Binding Moiety. *Mol Pharm* (2025) 22: 3423-3432. <https://doi.org/10.1021/acs.molpharmaceut.5c00291>

6. Wu CY, Lin JJ, Chang WY, Hsieh CY, Wu CC, Chen HS, et al. Development of theranostic active-targeting boron-containing gold nanoparticles for boron neutron capture therapy (BNCT). *Colloids Surf B Biointerfaces* (2019) 183: 110387. <https://doi.org/10.1016/j.colsurfb.2019.110387>

7. Huang W, Pan Y, Zhong T, He S, Qi Y,Huang Y. Near-infrared (10)B-BODIPY for precise guidance of tracer imaging and treatment in boron neutron capture therapy. *Chem Commun (Camb)* (2025) 61: 9079-9082. <https://doi.org/10.1039/d5cc01671a>

8. Coninx S, Kalot G, Godard A, Bodio E, Goze C, Sancey L, et al. Tailored hyaluronic acid-based nanogels as theranostic boron delivery systems for boron neutron cancer therapy. *Int J Pharm X* (2022) 4: 100134. <https://doi.org/10.1016/j.ijpx.2022.100134>

9. Kalot G, Godard A, Busser B, Pliquett J, Broekgaarden M, Motto-Ros V, et al. Aza-BODIPY: A New Vector for Enhanced Theranostic Boron Neutron Capture Therapy Applications. *Cells* (2020) 9: <https://doi.org/10.3390/cells9091953>

10. Zhong T, Yang Y, Pang M, Pan Y, Jing S, Qi Y, et al. Human Serum Albumin-Coated (10)B Enriched Carbon Dots as Targeted "Pilot Light" for Boron Neutron Capture Therapy. *Adv Sci (Weinh)* (2024) 11: e2406577. <https://doi.org/10.1002/advs.202406577>

11. Kim S, Mushtaq S, Lee KC, Park JA,Kim JY. (64)Cu-Labeled Boron-Containing Cyclic RGD Peptides for BNCT and PET Imaging. *ACS Med Chem Lett* (2024) 15: 344-348. <https://doi.org/10.1021/acsmedchemlett.4c00007>

12. Li J, Sun Q, Lu C, Xiao H, Guo Z, Duan D, et al. Boron encapsulated in a liposome can be used for combinational neutron capture therapy. *Nat Commun* (2022) 13: 2143. <https://doi.org/10.1038/s41467-022-29780-w>

13. Pulagam KR, Henriksen-Lacey M, K BU, Renero-Lecuna C, Kumar J, Charalampopoulou A, et al. In Vivo Evaluation of Multifunctional Gold Nanorods for Boron Neutron Capture and Photothermal Therapies. *ACS Appl Mater Interfaces* (2021) 13: 49589-49601. <https://doi.org/10.1021/acsami.0c17575>

14. Shi Y, Fu Q, Li J, Liu H, Zhang Z, Liu T, et al. Covalent Organic Polymer as a Carborane Carrier for Imaging-Facilitated Boron Neutron Capture Therapy. *ACS Appl Mater Interfaces* (2020) 12: 55564-55573. <https://doi.org/10.1021/acsami.0c15251>

15. Shi Y, Li J, Zhang Z, Duan D, Zhang Z, Liu H, et al. Tracing Boron with Fluorescence and Positron Emission Tomography Imaging of Boronated Porphyrin Nanocomplex for Imaging-Guided Boron Neutron Capture Therapy. *ACS Appl Mater Interfaces* (2018) 10: 43387-43395. <https://doi.org/10.1021/acsami.8b14682>

16. Cao J, Jin T, Shao S, Mao B,Feng J. Polymeric nanomaterials encapsulating fluorescent polyindole-nido- carborane: design, synthesis and biological evaluation. *Front Chem* (2024) 12: 1402640. <https://doi.org/10.3389/fchem.2024.1402640>

17. Ye M, Li B, Shi W, Liu H, Wang Y, Chen W, et al. Preparation and tumor-targeting evaluation of BS-CyP albumin nanoparticles modified with hyaluronic acid based on boron neutron capture therapy. *J Biomed Mater Res A* (2023) 111: 1176-1184. <https://doi.org/10.1002/jbm.a.37506>

18. Raskolupova VI, Wang M, Dymova MA, Petrov GO, Shchudlo IM, Taskaev SY, et al. Design of the New Closo-Dodecarborate-Containing Gemcitabine Analogue for the Albumin-Based Theranostics Composition. *Molecules* (2023) 28: <https://doi.org/10.3390/molecules28062672>

19. Popova T, Dymova MA, Koroleva LS, Zakharova OD, Lisitskiy VA, Raskolupova VI, et al. Homocystamide Conjugates of Human Serum Albumin as a Platform to Prepare Bimodal Multidrug Delivery Systems for Boron Neutron Capture Therapy. *Molecules* (2021) 26: <https://doi.org/10.3390/molecules26216537>

20. Kawai K, Nishimura K, Okada S, Sato S, Suzuki M, Takata T, et al. Cyclic RGD-Functionalized closo-Dodecaborate Albumin Conjugates as Integrin Targeting Boron Carriers for Neutron Capture Therapy. *Mol Pharm* (2020) 17: 3740-3747. <https://doi.org/10.1021/acs.molpharmaceut.0c00478>

21. Okada S, Nishimura K, Ainaya Q, Shiraishi K, Anufriev SA, Sivaev IB, et al. Development of a Gadolinium-Boron-Conjugated Albumin for MRI-Guided Neutron Capture Therapy. *Mol Pharm* (2023) 20: 6311-6318. <https://doi.org/10.1021/acs.molpharmaceut.3c00726>

22. Sforzi J, Lanfranco A, Stefania R, Alberti D, Bitonto V, Parisotto S, et al. A novel pH sensitive theranostic PLGA nanoparticle for boron neutron capture therapy in mesothelioma treatment. *Sci Rep* (2023) 13: 620. <https://doi.org/10.1038/s41598-023-27625-0>

23. Shanmugam M, Kuthala N, Kong X, Chiang CS,Hwang KC. Combined Gadolinium and Boron Neutron Capture Therapies for Eradication of Head-and-Neck Tumor Using Gd(10)B(6) Nanoparticles under MRI/CT Image Guidance. *JACS Au* (2023) 3: 2192-2205. <https://doi.org/10.1021/jacsau.3c00250>

24. Vares G, Jallet V, Matsumoto Y, Rentier C, Takayama K, Sasaki T, et al. Functionalized mesoporous silica nanoparticles for innovative boron-neutron capture therapy of resistant cancers. *Nanomedicine* (2020) 27: 102195. <https://doi.org/10.1016/j.nano.2020.102195>

25. Alberti D, Deagostino A, Toppino A, Protti N, Bortolussi S, Altieri S, et al. An innovative therapeutic approach for malignant mesothelioma treatment based on the use of Gd/boron multimodal probes for MRI guided BNCT. *J Control Release* (2018) 280: 31-38. <https://doi.org/10.1016/j.jconrel.2018.04.043>

26. Kuthala N, Vankayala R, Li YN, Chiang CS,Hwang KC. Engineering Novel Targeted Boron-10-Enriched Theranostic Nanomedicine to Combat against Murine Brain Tumors via MR Imaging-Guided Boron Neutron Capture Therapy. *Adv Mater* (2017) 29: <https://doi.org/10.1002/adma.201700850>

27. Alberti D, Protti N, Franck M, Stefania R, Bortolussi S, Altieri S, et al. Theranostic Nanoparticles Loaded with Imaging Probes and Rubrocurcumin for Combined Cancer Therapy by Folate Receptor Targeting. *ChemMedChem* (2017) 12: 502-509. <https://doi.org/10.1002/cmdc.201700039>

28. Mishiro K, Imai S, Ematsu Y, Hirose K, Fuchigami T, Munekane M, et al. RGD Peptide-Conjugated Dodecaborate with the Ga-DOTA Complex: A Preliminary Study for the Development of Theranostic Agents for Boron Neutron Capture Therapy and Its Companion Diagnostics. *J Med Chem* (2022) 65: 16741-16753. <https://doi.org/10.1021/acs.jmedchem.2c01586>

29. Sharma KS, Raju MS, Phapale S, Valvi SK, Dubey AK, Goswami D, et al. Multimodal Applications of Zinc Gallate-Based Persistent Luminescent Nanoparticles in Cancer Treatment: Tumor Margining, Diagnosis, and Boron Neutron Capture Therapy. *ACS Appl Bio Mater* (2022) 5: 3134-3145. <https://doi.org/10.1021/acsabm.2c00081>

30. Torresan V, Guadagnini A, Badocco D, Pastore P, Muñoz Medina GA, Fernàndez van Raap MB, et al. Biocompatible Iron-Boron Nanoparticles Designed for Neutron Capture Therapy Guided by Magnetic Resonance Imaging. *Adv Healthc Mater* (2021) 10: e2001632. <https://doi.org/10.1002/adhm.202001632>

31. Yamana K, Kawasaki R, Sanada Y, Tabata A, Bando K, Yoshikawa K, et al. Tumor-targeting hyaluronic acid/fluorescent carborane complex for boron neutron capture therapy. *Biochem Biophys Res Commun* (2021) 559: 210-216. <https://doi.org/10.1016/j.bbrc.2021.04.037>
